# MFMNet: Adaptive tri-layer graph pyramid with cross-scale attention router for synergistic drug prediction

**DOI:** 10.1371/journal.pone.0362586

**Published:** 2026-11-10

**Authors:** Lei Wang, Liping Sun, Jingwen Pan, Guangming Shao, Guokun Shi, Guanyu Zhang, Yucheng Shi, Li Jin

**Affiliations:** School of Medical Informatics Engineering, Anhui University of Chinese Medicine, Hefei, Anhui, China; Jinan University College of Pharmacy, CHINA

## Abstract

**Background and objective:**

Experimental screening of synergistic anticancer drug pairs is time-consuming, while effectively integrating complementary molecular graph representations with cell-line-specific information remains challenging for computational drug-synergy prediction.

**Methods:**

We developed MFMNet with a Tri-Layer Graph Pyramid (TGP) comprising GCN, GIN, and GAT branches for extracting complementary molecular graph representations. A Cross-Scale Attention Router (CSAR), implemented as an adaptive feature-wise gating mechanism, integrates the graph representations before they are combined with drug embeddings and cell-line transcriptomic features for synergistic drug-pair prediction.

**Results:**

In the initial five-fold cross-validation analysis, MFMNet achieved a mean five-fold AUROC of 0.9301 ± 0.0036. Under the stricter fold-matched evaluation protocol, the full MFMNet backbone achieved an AUROC of 0.9227 ± 0.0055 and a PR-AUC of 0.9127 ± 0.0084, with statistically significant improvements over XGBoost, DeepDDS-GCN, and DeepDDS-GAT after Holm correction.

**Conclusions:**

MFMNet provides a computational framework for synergistic drug-pair prediction by integrating complementary molecular graph representations with drug and cell-line features. The results support its use for computational prioritization of candidate synergistic drug combinations. All reported performance estimates refer to record-level five-fold cross-validation within the O'Neil dataset; generalization to entirely unseen drugs and cell lines requires further study.

## Introduction

The genotypic and phenotypic heterogeneity of cancer, including alterations in TP53, KRAS, and BRAF, variation in immune infiltration, and differences in drug metabolism, can limit the effectiveness of single-agent therapy [1]. Single-agent therapies may fail to suppress redundant signaling pathways; for example, the effects of EGFR inhibition in colorectal cancer can be attenuated by compensatory activation of MET signaling, contributing to treatment resistance and relapse [2]. The combination therapy targets complementary pathways [3]. In the MOSAIC trial, adding oxaliplatin to fluorouracil and leucovorin improved three-year disease-free survival from 72.9% to 78.2% [4]. Preclinical studies have shown that FAK inhibition can remodel an immunosuppressive tumor microenvironment and enhance responsiveness to PD-1 blockade [5]. In appropriate settings, combination strategies may also reduce treatment-related adverse effects by enabling complementary mechanisms or lower doses of individual agents [6,7].

However, not all drug combinations produce beneficial interactions, and some combinations may even be antagonistic [8]. Others are more toxic; overlapping myelosuppression between gemcitabine and docetaxel can amplify its toxicity. Consequently, correctly predicting the synergistic pair improves our chance of success [9].

Combination therapy has been widely explored across different areas of medicine, including modern oncology. Compared with monotherapy, appropriately designed drug combinations may improve therapeutic efficacy, reduce treatment-related adverse effects in suitable settings, and help delay or overcome drug resistance [10]. However, the number of possible drug combinations increases rapidly with the size of the candidate-drug set, making exhaustive experimental screening impractical. For a candidate set containing 1,000 drugs, the number of possible pairings can approach the order of 10^6, and even high-throughput screening platforms can evaluate only a fraction of these combinations [8]. Moreover, limited sample sizes and differences in experimental conditions can reduce reproducibility across studies.

Large-scale resources such as DrugComb and DrugCombDB provide extensive experimentally derived drug-combination data for computational modeling [11], while the Cancer Cell Line Encyclopedia (CCLE) contains gene-expression profiles across 947 cancer cell lines [12]. In parallel, high-throughput profiling initiatives such as the Genomics of Drug Sensitivity in Cancer (GDSC) project have enabled drug-response analyses across multiple cancer cell lines [13,14]. These resources have supported the development of computational approaches for drug-synergy prediction. Previous studies have explored different strategies for computational drug-synergy prediction. Preuer et al. developed DeepSynergy by integrating chemical descriptors of drug pairs with cancer-cell-line gene-expression profiles and reported a mean Pearson correlation coefficient of 0.73 between predicted and measured synergy values [15]. Celebi et al. integrated genomic, pharmacological, and drug-related features for the prediction of synergistic anticancer drug combinations [16]. Jiang et al. constructed a multimodal graph integrating drug–drug synergy, drug–target interaction, and protein–protein interaction networks and applied a graph convolutional network to predict cell-line-specific synergistic drug combinations [17].

Graph neural networks (GNNs) are well suited to molecular representation because they can directly model atoms as nodes and chemical bonds as edges within molecular graphs. Nguyen et al. proposed GraphDTA, which represents drug molecules as graphs and applies graph neural networks to learn molecular representations for drug–target binding-affinity prediction [18]. Ryu et al. developed DeepDDI, which uses drug structural similarity profiles and a deep neural network to predict multiple types of drug-drug interactions [19]. Zitnik et al. developed Decagon, a multirelational graph convolutional network integrating protein-protein, drug-protein, and drug-drug interaction information to predict polypharmacy side effects [20]. Ioannidis et al. constructed the Drug Repurposing Knowledge Graph (DRKG), integrating drugs, genes, diseases, and other biomedical entities for knowledge-graph-based drug-repurposing analyses [21]. Yang et al. evaluated a directed message-passing neural network for molecular property prediction and demonstrated the effectiveness of learned graph-based molecular representations across diverse public and industrial datasets [22].

These studies demonstrate the potential of graph neural networks to capture complex molecular topology and integrate heterogeneous biological information. However, effectively combining representations learned at different graph scales and coordinating molecular features with cell-line-specific information remain important challenges for drug-synergy prediction.

More recent graph- and attention-based models have further improved representation learning for drug-synergy prediction. DeepDDS represents drug chemical structures as molecular graphs and uses either a graph convolutional network or a graph attention network to derive drug embeddings, which are subsequently combined with cell-line gene-expression features for synergy prediction [23]. AttenSyn further employs graph neural networks together with attention-based pooling to capture interactive information between molecular representations of drug pairs [24]. HANSynergy constructs a heterogeneous biological graph and applies a heterogeneous graph attention network to integrate information from multiple biological entities and interactions [25]. In parallel, SynergyX uses mutual-attention mechanisms to model cross-modal interactions among drug and multi-omic cell-line features [26]. These approaches demonstrate the effectiveness of graph representation learning and attention mechanisms; however, they primarily emphasize a particular graph representation, heterogeneous biological-network modeling, drug-pair attention, or cross-modal attention. The coordinated extraction and integration of complementary molecular representations produced by different graph neural network operators at multiple graph scales remain less explicitly addressed.

To address this gap, we developed MFMNet, which combines a Tri-Layer Graph Pyramid (TGP) with a Cross-Scale Attention Router (CSAR). Rather than relying on a single graph encoder, the TGP combines complementary molecular representations generated by GCN, GIN, and GAT branches, while the CSAR adaptively reweights the concatenated graph features before their integration with drug embeddings and cell-line transcriptomic features for synergistic drug-pair prediction (Fig 1). Thus, the architectural distinction of MFMNet lies in the coordinated use of complementary graph encoders and adaptive feature routing rather than in the use of attention alone. The performance of MFMNet was evaluated using five-fold cross-validation and further examined through fold-matched comparisons with principal baseline models under a stricter outer-test evaluation protocol. Controlled leave-one-branch-out and fusion ablations, together with branch-level representation and prediction diagnostics, were further used to examine the contribution and complementarity of the TGP and CSAR components.

**Fig 1:**
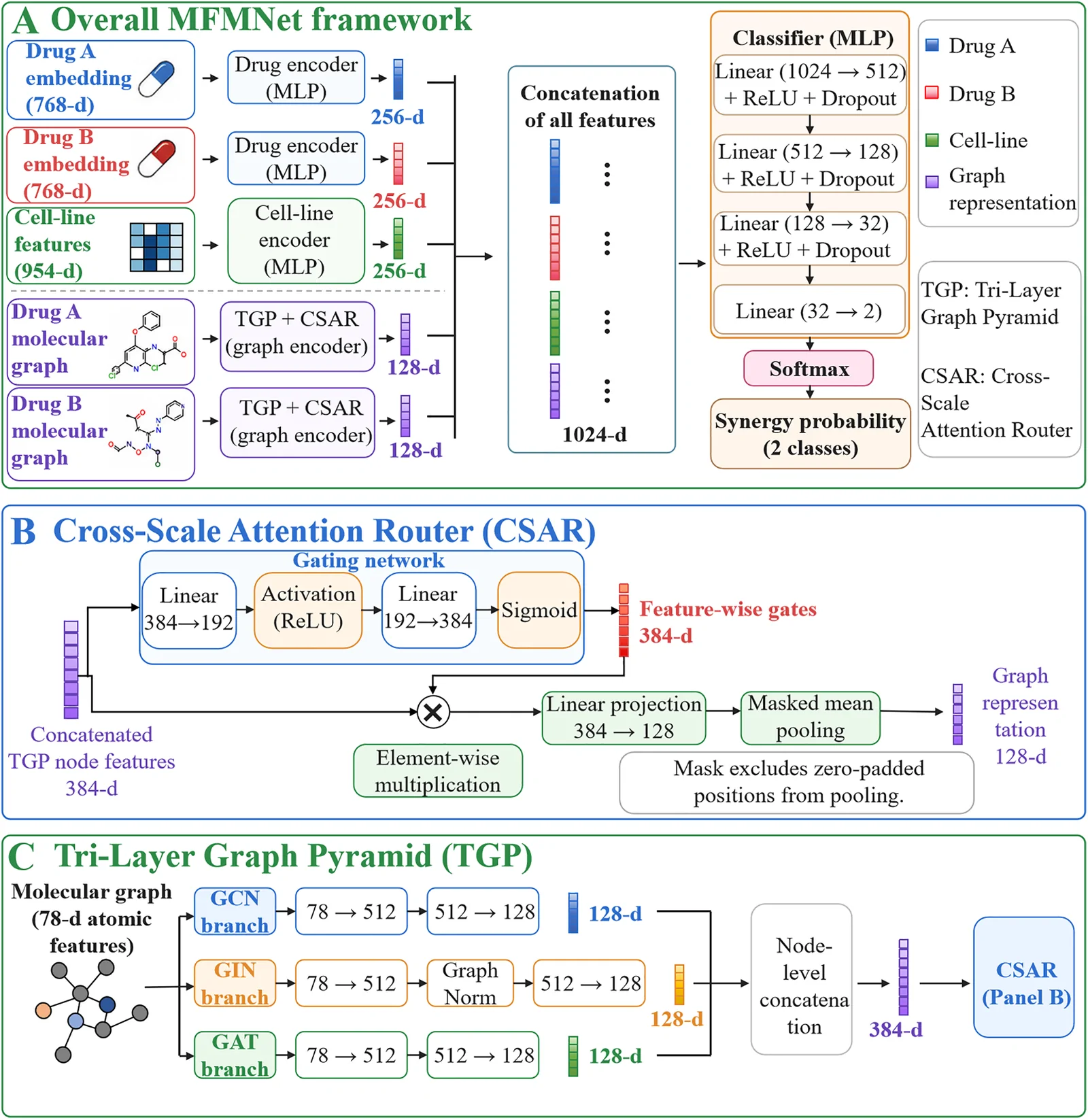
Overall architecture of MFMNet. (A) Multimodal feature encoding and classification. (B) Cross-Scale Attention Router (CSAR). (C) Tri-Layer Graph Pyramid (TGP).

## Materials and methods

### Data source and label definition

The drug-combination dataset used in the implemented experiments was obtained from the publicly available data released with DeepDDS [23]. The analyzed dataset contained 13,243 drug-pair–cell-line records involving 38 unique drugs and 31 cancer cell lines. Each record contained two drug representations, the corresponding cancer-cell-line features, a drug-combination synergy score, and a binary label. The supplied dataset defined records with synergy scores greater than 10 as synergistic and records with synergy scores below 0 as non-synergistic. Records with synergy scores between 0 and 10 were not included in the analyzed dataset. The resulting dataset comprised 6,188 synergistic records and 7,055 non-synergistic records, corresponding to 46.7% and 53.3% of the analyzed samples, respectively. No additional resampling or class weighting was applied. No additional negative samples were synthetically generated during model training. Negative samples corresponded to the records labeled as non-synergistic in the supplied dataset according to the synergy-score criterion described above. Because the positive and negative classes represented 46.7% and 53.3% of the analyzed records, respectively, the original class distribution was retained without oversampling, under sampling, or class-weighted loss.

### Ethics statement

This study was based exclusively on publicly available cell-line, gene-expression, and drug-response datasets and involved no recruitment of human participants, collection of human specimens, or animal experiments by the authors. No personally identifiable information was accessed or analyzed. Therefore, institutional ethics approval and informed consent were not required for this secondary computational analysis.

### Data preprocessing

For each drug-pair–cell-line record, the SMILES strings of the two drugs were converted into molecular graphs using RDKit [27]. Each atom was represented by a 78-dimensional feature vector encoding atom type, degree, total number of attached hydrogen atoms, implicit valence, and aromaticity. The atom-feature vector was normalized by the sum of its elements, and each molecular graph was represented using a fixed maximum of 100 atomic positions. Unused positions were zero-padded and explicitly marked by a validity mask so that padded positions were excluded from graph normalization and graph-level pooling. Chemical bonds were represented as bidirectional graph edges. In parallel, the supplied precomputed drug-pair embedding file contained 1,536 features per record, which were divided into two 768-dimensional vectors corresponding to the two drugs. The 954-dimensional cell-line transcriptomic profile associated with each record was read directly from the supplied dataset.

### Hyperparameter settings

The 13,243 records were partitioned into five outer folds using a fixed random seed, with each record used as an outer-test sample once. For each outer fold, 10% of the remaining records were selected by stratified sampling as the validation set, and the remaining records were used for model training. Model selection and early stopping were based exclusively on the validation AUROC, and the corresponding outer-test fold was evaluated only after the best-performing model checkpoint had been determined. The model was trained for a maximum of 300 epochs with a batch size of 64 using the Adam optimizer, an initial learning rate of 5 × 10 ⁻ ⁴, cross-entropy loss, an L2 regularization coefficient of 1 × 10^−5^, and a dropout rate of 0.3. A ReduceLROnPlateau scheduler reduced the learning rate by a factor of 0.5 when the training loss did not improve for 10 epochs. Training was stopped when the validation AUROC did not improve by more than 1 × 10 ⁻ ⁴ for 20 consecutive epochs, and the checkpoint with the highest validation AUROC was retained for evaluation on the corresponding outer-test fold. The hyperparameter configuration was kept fixed across all five outer folds, and no fold-specific hyperparameter tuning was performed using the outer-test data. A fixed base random seed was used for reproducible data partitioning and model training. The same hyperparameter configuration was applied to all outer folds so that model selection remained independent of the corresponding outer-test partitions. Hyperparameter tuning and model selection were therefore performed exclusively on the training and validation data of each outer fold, and no information from the outer-test folds—including records, labels, or summary statistics—was used at any stage of model development; each outer-test fold was evaluated exactly once, after the final checkpoint had been frozen.

### GCN

The Tri-Layer Graph Pyramid (TGP) comprises three parallel graph-encoding branches based on GCN, GIN, and GAT. The three branches process the same atomic feature input independently, and their outputs are concatenated before adaptive fusion by the CSAR.

The graph convolutional network (GCN) branch [28] processes each molecular graph through two graph convolutional layers. The first GCN layer maps the 78-dimensional atomic features to 512-dimensional node representations, followed by a ReLU activation and dropout with a rate of 0.3. The second GCN layer further maps the node representations from 512 to 128 dimensions. The graph convolution operation is expressed as


$$\begin{array}{c} {𝐇}^{\left(l+\text{1}\right)}={\hat{𝐃}}^{-\frac{\text{1}}{\text{2}}}\hat{𝐀}{\hat{𝐃}}^{-\frac{\text{1}}{\text{2}}}{𝐇}^{\left(l\right)}{𝐖}^{\left(l\right)} \end{array}$$ (1)

Where $\hat{𝐀}=A+I$ is the adjacency matrix with added self-loops, $\hat{𝐃}$ is the corresponding degree matrix, ${𝐇}^{\left(l\right)}$ denotes the feature matrix of the l -th layer. and ${𝐖}^{\left(l\right)}$ is the trainable transformation matrix. The same GCN branch is applied to the two drug molecular graphs, producing a 128-dimensional node representation for each atom. These representations are subsequently combined with the node representations generated by the GIN and GAT branches for adaptive graph-feature fusion.

### GIN

The Graph Isomorphism Network (GIN) branch [29] processes each molecular graph through two GIN convolutional layers. In the first layer, neighborhood features are aggregated and transformed by a multilayer perceptron with dimensions of 78–512–512. The resulting 512-dimensional node representations are processed by a ReLU activation, GraphNorm, and dropout with a rate of 0.3 before being passed to the second GIN layer. The second GIN layer employs a multilayer perceptron with dimensions of 512–128–128 and produces a 128-dimensional representation for each atom. The neighborhood aggregation used in the implemented GIN branch can be expressed as


$$\begin{array}{c} h_{v}^{\left(l+\text{1}\right)}=MLP^{\left(l\right)}(h_{v}^{\left(l\right)}+\sum_{u\in N(v)}h_{u}^{\left(l\right)}) \end{array}$$ (2)

Where $h_{v}^{\left(l\right)}$ denotes the representation of atom v at layer l, v denotes the set of neighboring atoms of v, and $MLP^{\left(l\right)}$ denotes the multilayer perceptron associated with the corresponding GIN convolutional layer. The same GIN branch is applied to both drug molecular graphs, and its 128-dimensional node representations are subsequently combined with those generated by the GCN and GAT branches.

### GAT

The graph attention branch was implemented using two GAT convolutional layers (GATv2Conv) [30]. The first layer maps the 78-dimensional atomic features to 256 dimensions using two attention heads. The outputs of the two heads are concatenated to form a 512-dimensional node representation, followed by a ReLU activation and dropout with a rate of 0.3. The second GAT layer uses a single attention head to transform the 512-dimensional representation into a 128-dimensional representation for each atom. The attention coefficient between atom i and its neighboring atom j is expressed as


$$\begin{array}{c} \alpha_{ij}=\frac{exp(LeakyReLU({\overset{⇀}{a}}^{T}\Theta_{s}{𝐡}_{i}+\Theta_{t}{𝐡}_{j}]))}{\sum_{k\in N(i)}exp(LeakyReLU({\overset{⇀}{a}}^{T}[\Theta_{s}{𝐡}_{i}+\Theta_{t}{𝐡}_{k}]))} \end{array}$$ (3)

Where ij denotes the normalized attention coefficient from atom j to atom i, ${𝐡}_{i}$ and ${𝐡}_{j}$ denote their node representations, $\Theta_{s}$ and $\Theta_{t}$ are trainable linear transformations, and ${\overset{⇀}{a}}^{T}$ is the trainable attention vector. Molecular bonds define the neighborhood connectivity of the molecular graph used by the graph-attention layers. The same GAT branch is applied to both drug molecular graphs, and the resulting 128-dimensional node representations are subsequently combined with those generated by the GCN and GIN branches.

### Graph normalization

GraphNorm was applied to the 512-dimensional node representations generated by the first GIN convolutional layer. Specifically, the output of the first GIN layer was processed by a ReLU activation followed by GraphNorm [31] computed over valid atomic positions only and dropout before being passed to the second GIN layer; zero-padded positions were excluded from the normalization statistics.

### Feature processing module

Drug Molecular Encoding: In addition to the molecular-graph representations generated by the GCN, GIN, and GAT branches, each drug was represented by a 768-dimensional embedding. The drug embedding was processed by two fully connected layers with dimensions of 768–512–256. Batch normalization and ReLU activation were applied after each fully connected layer, and dropout with a rate of 0.2 was applied after the first activation.

Cell Line Encoding: Each cell line was represented by a 954-dimensional transcriptomic feature vector. The cell-line features were encoded by two fully connected layers with dimensions of 954–512–256. Batch normalization and ReLU activation were applied after each fully connected layer, with dropout at a rate of 0.3 applied between the two layers.

### Adaptive feature fusion module

For each atom, the 128-dimensional node representations generated by the GCN, GIN, and GAT branches were concatenated to form a 384-dimensional feature vector. The three branch outputs were aligned at the node level and concatenated along the feature dimension; CSAR then generated feature-wise gates from the concatenated representation, applied these gates to the same representation, projected the weighted features to 128 dimensions, and subsequently performed graph-level mean pooling. The Cross-Scale Attention Router (CSAR) was implemented as an adaptive feature-wise gating network that first transformed the concatenated representation from 384 to 192 dimensions and then back to 384 dimensions. A sigmoid activation generated feature-wise weights, which were applied to the concatenated representation by element-wise multiplication. The weighted representation was subsequently projected from 384 to 128 dimensions. The adaptive weighting and fusion operations can be expressed as


$$\begin{array}{c} w=Sigmoid(W_{\text{2}}ReLU(W_{\text{1}}z+b_{\text{1}})+b_{\text{2}}) \end{array}$$ (4)

$$\begin{array}{c} f=W_{p}(z⊙w)+b_{p} \end{array}$$ (5)

Where z denotes the concatenated GCN, GIN, and GAT node representation, denotes the feature-wise gating weights, ⊙ denotes element-wise multiplication, and f denotes the resulting 128-dimensional fused node representation. After adaptive fusion, masked mean pooling was applied over the valid atomic positions of each drug graph, excluding zero-padded positions, to obtain a 128-dimensional graph-level representation for each drug.

### Classification module

For classification, the 256-dimensional embeddings of the two drugs, the 256-dimensional cell-line representation, and the two 128-dimensional graph-level drug representations were concatenated to form a 1,024-dimensional feature vector. The combined representation was passed through three hidden fully connected layers with dimensions of 1,024–512–128–32, followed by a two-unit output layer for binary classification. Batch normalization was applied after the 512- and 128-dimensional fully connected layers, and ReLU activation was used after each hidden layer. Dropout with a rate of 0.3 was applied after the 512-, 128-, and 32-dimensional hidden representations. The two output logits were converted into class probabilities using the softmax function, with the positive-class probability used for performance evaluation.

### Statistical analysis

Performance metrics across the five outer-test folds were summarized as the mean and standard deviation, with 95% confidence intervals calculated using Student’s t distribution. All baseline models and controlled ablation variants were evaluated using identical outer-test fold assignments to enable fold-matched comparisons. Differences in AUROC and PR-AUC between MFMNet and each baseline model were evaluated using two-sided paired t-tests, with Holm adjustment applied separately within the AUROC and PR-AUC baseline-comparison families. For the primary controlled ablation analysis, the full MFMNet backbone was compared with five controlled variants: removal of GCN, removal of GIN, removal of GAT, replacement of CSAR with unweighted mean fusion, and replacement of CSAR with simple concatenation followed by linear projection. Two-sided paired t-tests were performed on identical outer-test folds, with Holm adjustment applied separately to the five AUROC comparisons and the five PR-AUC comparisons. GCN-only, GIN-only, and GAT-only models were analyzed separately as descriptive branch-complementarity diagnostics and were not included in the primary Holm correction families. A two-sided Holm-adjusted p-value below 0.05 was considered statistically significant for multiple comparisons.

#### Additional external validation.

To assess cross-dataset generalization, additional external evaluations were performed using the FRIEDMAN and NCI-ALMANAC drug-combination datasets. Exact drug-pair–cell-line records overlapping with the O’Neil dataset were excluded before evaluation. For FRIEDMAN, records with Loewe synergy scores greater than 10 were classified as synergistic and those with scores below 0 as non-synergistic, yielding 39 classifiable records involving 10 drugs and 5 cell lines. For NCI-ALMANAC, replicate observations for the same drug-pair–cell-line combination were averaged, and the same classification thresholds were applied to the resulting ComboScore values, yielding 864 classifiable records involving 22 drugs and 8 cell lines. Because the supplied 768-dimensional auxiliary drug embeddings were not consistently transferable to external records, external evaluation used the embedding-independent MFMNet configuration while retaining the molecular-graph branches, CSAR, cell-line encoder, and classifier. Five-fold-specific checkpoints selected using only the O’Neil training and validation data were evaluated with equal-weight probability averaging. A fixed classification threshold of 0.5 was used, and 95% confidence intervals for AUROC and PR-AUC were estimated using 5,000 bootstrap replicates.

## Results

### Top 10 predicted synergistic drug pairs by MFMNet: F1-score performance and literature support

Table 1 lists the top 10 drug pairs predicted by MFMNet, ranked by F1 score. Publicly available studies involving the same drug combination were identified for six of the predicted pairs, whereas no direct published evidence for the exact drug combination was identified for the remaining four pairs. The Publications column summarizes available reports involving the corresponding drug combinations and provides literature context for the predicted pairs. Among the high-scoring drug pairs listed in Table 1, VINBLASTINE–L778123 achieved an F1 score of 0.982, with an accuracy of 0.966, precision of 0.964, and recall of 1.000 in the pair-specific evaluation. These results indicate strong classification performance for VINBLASTINE–L778123 in the evaluated samples and are consistent with its high ranking among the predicted synergistic drug pairs.

**Table 1:** Top 10 predicted synergistic drug pairs ranked by F1-score.

Drug A	Drug B	Samples	ACC	AUPR	AUC	F1	Precision	Recall	Publications
VINBLASTINE	L778123	29	0.966	0.994	0.926	0.982	0.964	1	No direct evidence identified
MITOMYCINC	DASATINIB	29	0.966	1	1	0.98	1	0.962	No direct evidence identified
TOPOTECAN	BEZ-235	25	0.96	0.998	0.958	0.979	1	0.958	No direct evidence identified
MK-2206	SORAFENIB	25	0.96	1	1	0.978	0.957	1	[32]
SUNITINIB	DASATINIB	24	0.958	0.998	0.957	0.978	1	0.957	[33]
DOXORUBICIN	DASATINIB	23	0.957	0.998	0.976	0.977	0.955	1	[34]
VINORELBINE	BEZ-235	27	0.963	1	1	0.977	0.955	1	[35]
AZD1775	DASATINIB	42	0.952	0.997	0.957	0.974	0.974	0.974	[36]
ETOPOSIDE	ZOLINZA	22	0.955	0.98	0.917	0.973	0.947	1	[37]
L778123	ERLOTINIB	22	0.955	0.99	0.944	0.971	1	0.944	No direct evidence identified

### Visualization of learned atomic-feature representations

To examine changes in atomic-feature representations after model training, three molecular comparisons were visualized in Fig 2: VINBLASTINE–L778123, L778123–L778123, and VINBLASTINE–VINBLASTINE. All heatmaps were computed from the trained first-fold model and, as a reference, from the same architecture before training (random initialization with the identical seed). Compared with the pre-training representations, the post-training heatmaps showed globally more aligned atomic-feature similarities (for example, the mean off-diagonal similarity of the L778123 self-comparison increased from 0.665 to 0.952), while chemically distinct atoms—such as a subset of VINBLASTINE atoms forming clearly separated rows and columns—remained differentiated from the aligned background. This pattern indicates that training reshaped the atomic-feature space toward task-oriented alignment while preserving separability for structurally distinct atoms.

**Fig 2:**
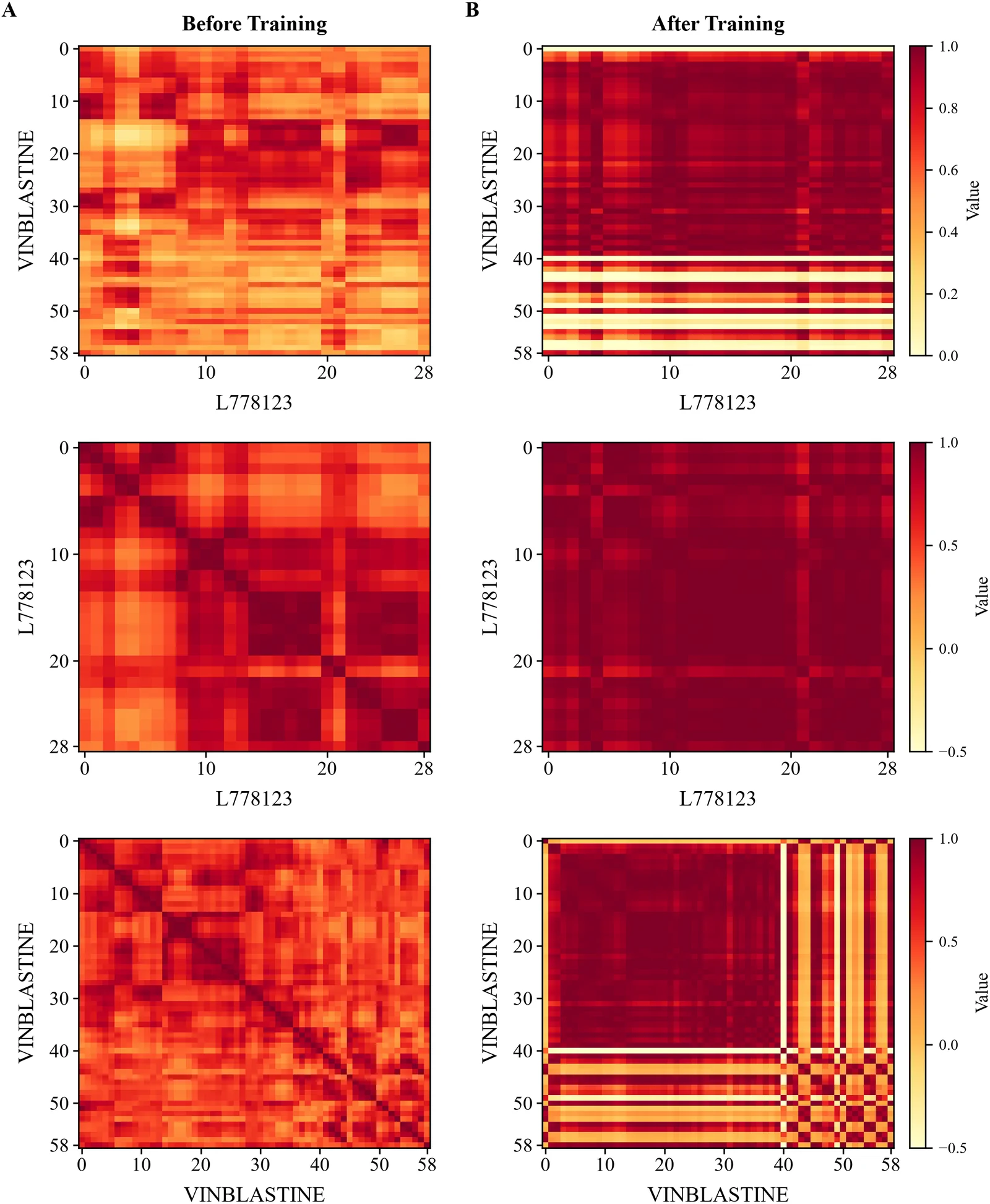
Atomic-feature similarity heatmaps for VINBLASTINE and L778123 before and after MFMNet training.

### Multi-dimensional performance evaluation of the model

Fig 3 summarizes the predictive performance of MFMNet and DeepDDS in the initial five-fold cross-validation analysis. Fig 3A shows the prediction-score distributions for positive and negative samples, with MFMNet exhibiting greater separation between the two classes in the displayed distributions. In Fig 3B, the displayed precision–recall curves yielded areas under the curve of 0.8875 for MFMNet and 0.8636 for DeepDDS. These values characterize the curves shown in this panel and are distinct from the fold-averaged PR-AUC values used in the fold-matched statistical analysis. Fig 3C shows the five-fold-specific ROC curves of MFMNet in the initial five-fold cross-validation analysis, with AUROC values ranging from 0.9251 to 0.9334. The area under the interpolated mean ROC curve was 0.929, whereas the arithmetic mean of the five-fold-specific AUROC values was 0.9301 ± 0.0036. Under the stricter fold-matched evaluation protocol using inner-validation-based model selection, the full MFMNet backbone achieved an AUROC of 0.9227 ± 0.0055 and a PR-AUC of 0.9127 ± 0.0084 across the five outer-test folds. The latter results were used for the formal fold-matched comparisons with the principal baseline models reported below.

**Fig 3:**
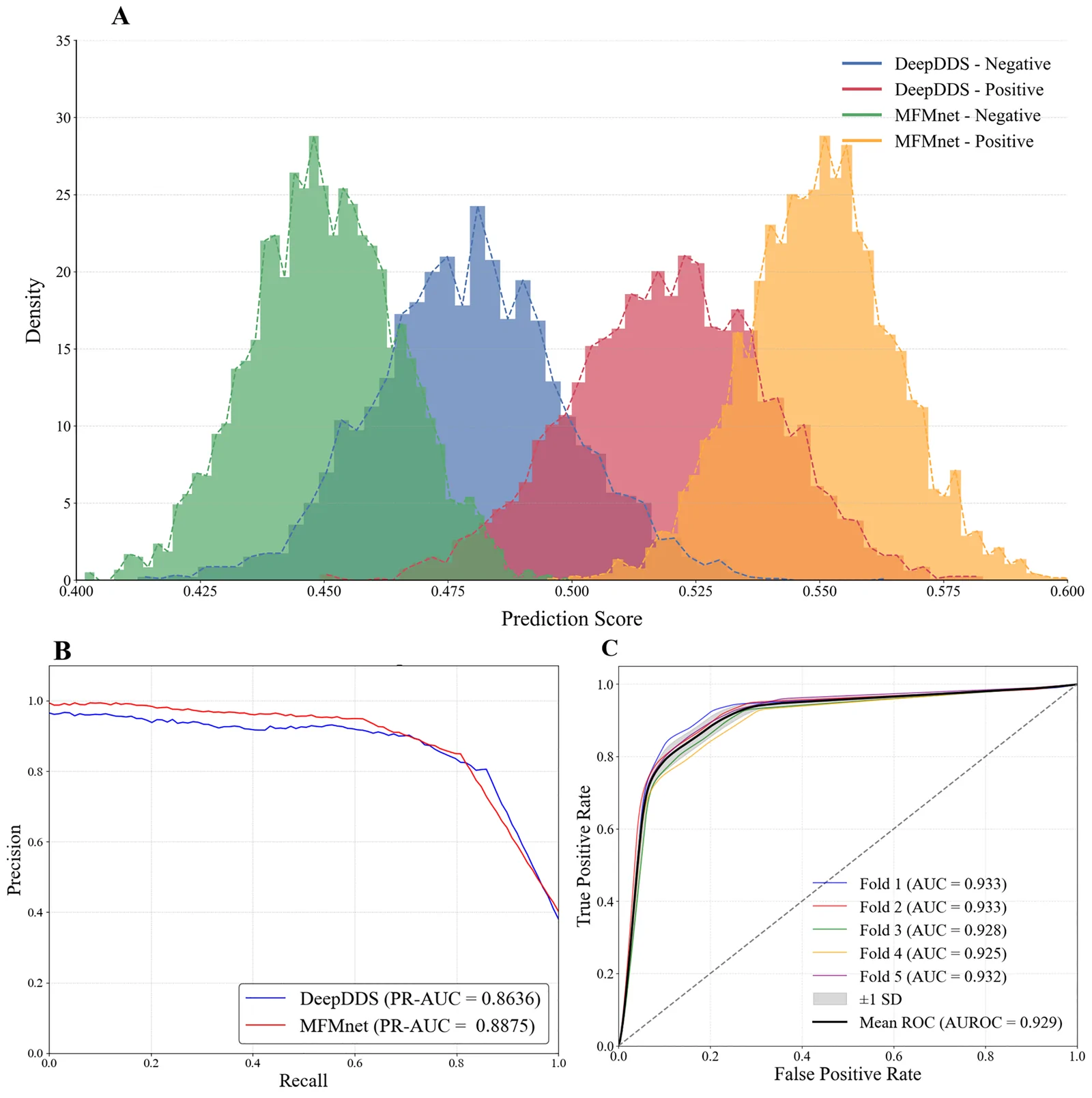
Performance visualization of MFMNet and DeepDDS in the initial five-fold cross-validation analysis. (A) Prediction-score distributions for positive and negative samples. (B) Precision–recall curves of MFMNet and DeepDDS. (C) Five-fold-specific receiver operating characteristic curves of MFMNet.

### Cell-line-specific performance comparison between MFMNet and DeepDDS

Fig 4 compares the cell-line-specific precision and mean squared error (MSE) of MFMNet and DeepDDS. MFMNet showed lower MSE for most of the cell lines displayed in Fig 4B, including A2780 and HT29, indicating smaller prediction errors in these cell-line-specific evaluations. The precision results in Fig 4A further show that the relative performance of the two models varied across individual cell lines. Therefore, the cell-line-specific analysis suggests that the performance difference between MFMNet and DeepDDS was not uniform across all cell lines, although MFMNet showed lower prediction error for most of the displayed cell lines.

**Fig 4:**
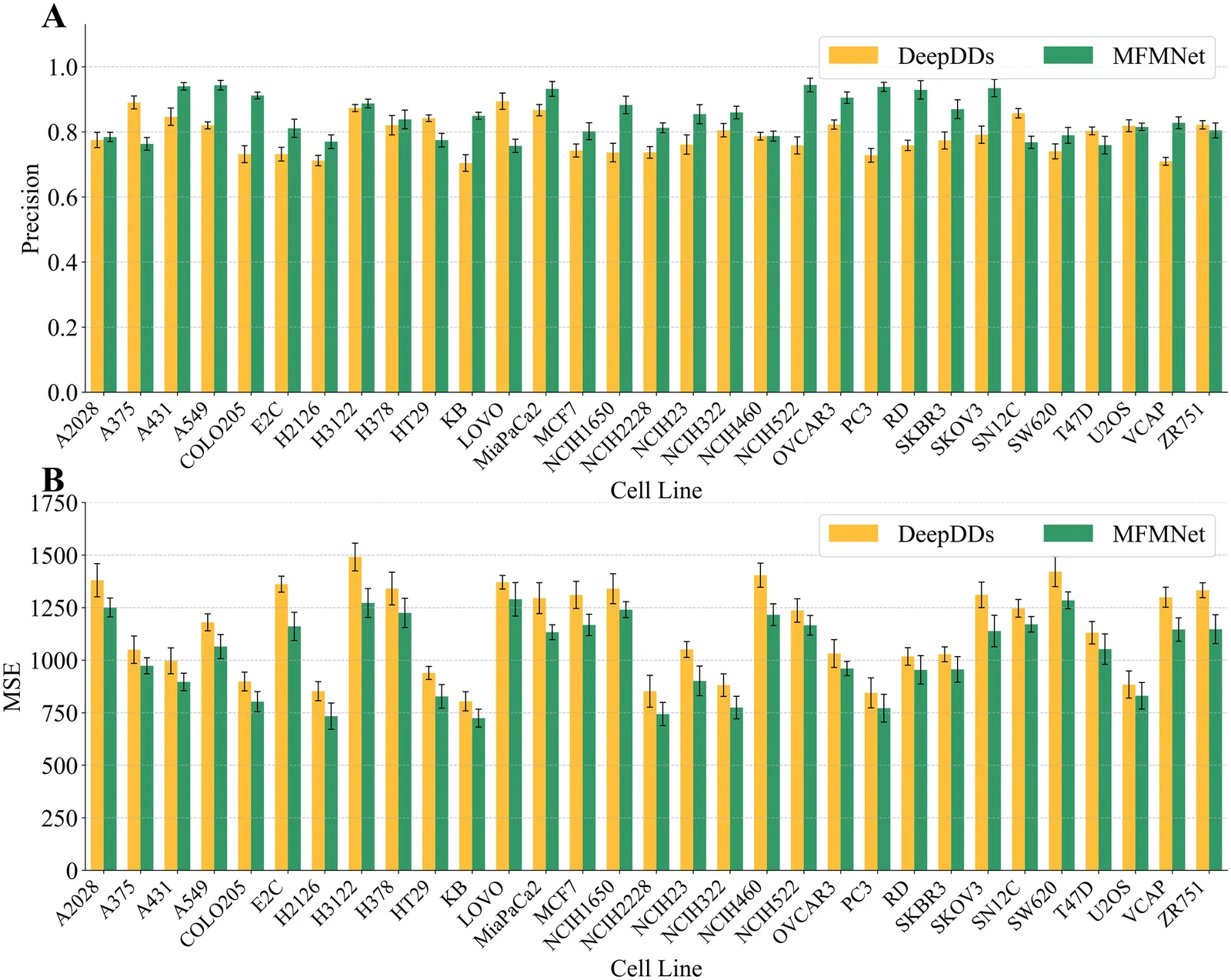
Cell-line-specific performance comparison between MFMNet and DeepDDS. (A) Precision across individual cell lines. (B) Mean squared error (MSE) across individual cell lines.

### Multi-metric comparative experiments using state-of-the-art methods

Table 2 summarizes the multi-metric comparison obtained in the initial five-fold cross-validation analysis, in which MFMNet showed competitive performance across the evaluated models and metrics. To further assess whether the observed differences were reproducible under a stricter and uniform evaluation protocol, MFMNet and the principal baseline models were re-evaluated using identical five outer-test fold assignments.

**Table 2:** Multi-metric comparison of MFMNet and baseline models in the initial five-fold cross-validation analysis.

Model	AUC	PR_AUC	ACC	BACC	Precision	TPR	KAPPA
**MFMNet** **(Ours)**	**0.9301 ± 0.0036**	**0.9334 ± 0.023**	**0.8537 ± 0.024**	**0.85281 ± 0.053**	**0.8462 ± 0.065**	**0.8394 ± 0.036**	**0.7158 ± 0.025**
DEEPDDS (GAT)	0.9273 ± 0.021	0.9218 ± 0.014	0.8457 ± 0.016	0.8532 ± 0.015	0.8469 ± 0.012	0.8471 ± 0.015	0.7124 ± 0.021
DEEPDDS (GCN)	0.9286 ± 0.021	0.9214 ± 0.015	0.8492 ± 0.025	0.8479 ± 0.026	0.8513 ± 0.014	0.8398 ± 0.013	0.7035 ± 0.022
XGBoost	0.9164 ± 0.012	0.9219 ± 0.016	0.8275 ± 0.015	0.8341 ± 0.035	0.8368 ± 0.012	0.8392 ± 0.035	0.6817 ± 0.013
Random Forest	0.8573 ± 0.023	0.8469 ± 0.016	0.7684 ± 0.014	0.7732 ± 0.013	0.7815 ± 0.024	0.7368 ± 0.014	0.5473 ± 0.042
GBM	0.8536 ± 0.021	0.8472 ± 0.013	0.7628 ± 0.013	0.7584 ± 0.015	0.7693 ± 0.022	0.7421 ± 0.016	0.5316 ± 0.056
AdaBoost	0.8275 ± 0.016	0.8268 ± 0.025	0.7362 ± 0.015	0.7419 ± 0.045	0.7384 ± 0.052	0.7216 ± 0.013	0.4813 ± 0.045
MLP	0.6479 ± 0.023	0.6283 ± 0.064	0.5571 ± 0.063	0.5634 ± 0.026	0.5397 ± 0.016	0.5276 ± 0.022	0.1238 ± 0.043
SVM	0.5762 ± 0.024	0.5584 ± 0.011	0.5369 ± 0.013	0.5391 ± 0.024	0.5362 ± 0.013	0.5073 ± 0.012	0.0764 ± 0.013
AuDNNsynergy	0.9137 ± 0.013	0.6274 ± 0.014	0.9276 ± 0.003	NA	0.7183 ± 0.026	NA	0.5082 ± 0.046

To provide a stricter statistical comparison, MFMNet, XGBoost, DeepDDS-GCN, and DeepDDS-GAT were re-evaluated using identical outer-test fold assignments, with model selection performed exclusively on the corresponding inner validation subsets. Under the revised fold-matched evaluation protocol, the full MFMNet backbone achieved a mean AUROC of 0.9227 ± 0.0055 and a mean PR-AUC of 0.9127 ± 0.0084 across the five outer-test folds. As shown in Table 3, MFMNet achieved the highest mean AUROC and PR-AUC among the four evaluated models and positive mean paired differences relative to XGBoost, DeepDDS-GCN, and DeepDDS-GAT for both metrics. Two-sided paired t-tests followed by Holm correction showed that all six-fold-matched comparisons remained statistically significant at the adjusted p < 0.05 level. In particular, MFMNet significantly outperformed both DeepDDS variants on both metrics under Holm-adjusted paired t-tests: p = 0.0099 (AUROC) and 0.0075 (PR-AUC) versus DeepDDS-GCN, and p = 0.0130 (AUROC) and 0.0332 (PR-AUC) versus DeepDDS-GAT.

**Table 3:** Fold-matched statistical comparisons between the MFMNet backbone and baseline models.

Comparison	Metric	MFMNet backbone	Baseline	Mean paired difference	95% CI of difference	Paired t-test p	Holm-adjusted p
MFMNet vs DeepDDS-GCN	AUROC	**0.9227 ± 0.0055**	0.8968 ± 0.0079	+0.0259	0.0144–0.0374	0.0033	**0.0099**
MFMNet vs DeepDDS-GAT	AUROC	**0.9227 ± 0.0055**	0.8911 ± 0.0142	+0.0316	0.0110–0.0521	0.0130	**0.0130**
MFMNet vs XGBoost	AUROC	**0.9227 ± 0.0055**	0.9149 ± 0.0028	+0.0078	0.0037–0.0120	0.0062	**0.0125**
MFMNet vs DeepDDS-GCN	PR-AUC	**0.9127 ± 0.0084**	0.8843 ± 0.0130	+0.0284	0.0168–0.0401	0.0025	**0.0075**
MFMNet vs DeepDDS-GAT	PR-AUC	**0.9127 ± 0.0084**	0.8805 ± 0.0154	+0.0322	0.0096–0.0549	0.0168	**0.0332**
MFMNet vs XGBoost	PR-AUC	**0.9127 ± 0.0084**	0.9054 ± 0.0058	+0.0073	0.0022–0.0125	0.0166	**0.0332**

Values are reported as mean ± standard deviation across five identical outer-test folds. Mean paired differences were calculated as MFMNet backbone minus the corresponding baseline. Two-sided paired t-tests were performed using the fold-matched AUROC and PR-AUC values, and 95% confidence intervals were calculated for the paired differences. To account for multiple comparisons, p-values were adjusted using the Holm method separately within the AUROC and PR-AUC comparison families. A two-sided Holm-adjusted p-value below 0.05 was considered statistically significant. Bold values in the Holm-adjusted p column indicate statistically significant comparisons after multiple-comparison correction.

### Fold-matched performance comparison with principal baseline models

The fold-matched comparison of MFMNet with the principal baseline models is further illustrated in Fig 5. Across the five identical outer-test folds, MFMNet achieved the highest mean AUROC and PR-AUC among MFMNet, XGBoost [38], DeepDDS-GCN, and DeepDDS-GAT, whereas XGBoost achieved a slightly higher mean precision. The relatively small standard deviations observed for the primary discrimination metrics indicate that the performance estimates were generally stable across the five data partitions. Together with the paired statistical comparisons in Table 3, these results show that MFMNet maintained a consistent performance advantage over the two DeepDDS variants and a modest advantage over XGBoost in AUROC and PR-AUC under the uniform fold-matched evaluation protocol.

**Fig 5:**
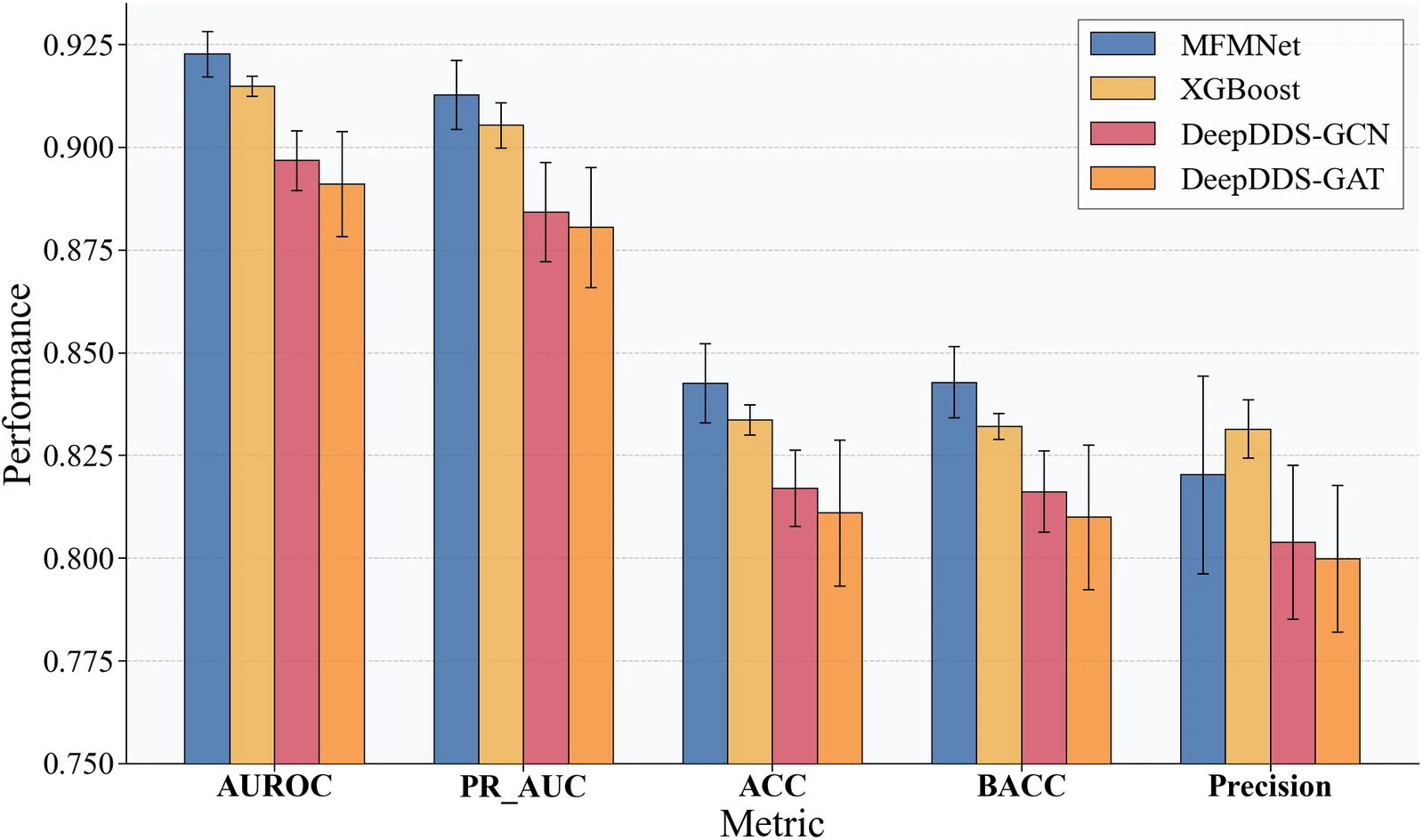
Fold-matched performance comparison of MFMNet and principal baseline models. Mean AUROC, PR-AUC, ACC, BACC, and precision are shown for MFMNet, XGBoost, DeepDDS-GCN, and DeepDDS-GAT across five identical outer-test folds. Error bars represent the standard deviation across the five folds.

### Controlled ablation analysis of TGP and CSAR

To quantify the contributions of the TGP branches and CSAR, we performed a controlled ablation analysis using the same five outer-test folds, inner-validation procedure, fold-specific initialization reference, optimization budget, and stopping rule. The full model achieved an AUROC of 0.9227 ± 0.0055 and a PR-AUC of 0.9127 ± 0.0084. Removing GCN or GIN yielded small decreases in mean AUROC to 0.9213 ± 0.0039 and 0.9206 ± 0.0024, respectively, whereas removal of GAT produced essentially unchanged AUROC (0.9229 ± 0.0032). Replacing CSAR with unweighted mean fusion reduced AUROC to 0.9192 ± 0.0041 and PR-AUC to 0.9099 ± 0.0054, while simple concatenation followed by linear projection yielded an AUROC of 0.9182 ± 0.0010 and a PR-AUC of 0.9102 ± 0.0044. The mean paired AUROC differences in favor of the full model were 0.0035 for mean fusion and 0.0045 for simple concatenation. None of the five primary AUROC or PR-AUC comparisons reached statistical significance after Holm correction.

Single-branch diagnostic analyses further examined whether the GCN, GIN, and GAT branches captured redundant or complementary information. Pairwise prediction disagreement rates ranged from approximately 8.7% to 9.1% across the three branch pairs. Each graph encoder also uniquely classified a subset of test samples correctly, averaging 50.8 samples per fold for GCN, 44.4 for GIN, and 52.0 for GAT. The descriptive any-branch oracle accuracy was 0.9054 ± 0.0029, compared with individual single-branch accuracies of approximately 0.840–0.844. Pairwise cosine similarities between the graph-level branch representations were also low overall. These findings indicate that the GCN, GIN, and GAT branches learned substantially non-redundant representations and exhibited partially complementary prediction patterns, although their marginal contributions to aggregate AUROC were heterogeneous, as shown in Table 4.

**Table 4:** Controlled ablation analysis of the TGP branches and CSAR fusion strategy.

Variant	AUROC mean ± SD	ΔAUROC vs full	Holm-adjusted p	PR-AUC mean ± SD	ΔPR-AUC vs full	Holm-adjusted p
Full MFMNet	0.9227 ± 0.0055	—	—	0.9127 ± 0.0084	—	—
w/o GCN	0.9213 ± 0.0039	+0.0014	1.0000	0.9141 ± 0.0046	−0.0014	1.0000
w/o GIN	0.9206 ± 0.0024	+0.0021	0.7579	0.9120 ± 0.0064	+0.0008	1.0000
w/o GAT	0.9229 ± 0.0032	−0.0002	1.0000	0.9147 ± 0.0047	−0.0020	1.0000
w/o CSAR, mean fusion	0.9192 ± 0.0041	+0.0035	0.7579	0.9099 ± 0.0054	+0.0028	1.0000
Simple concatenation + projection	0.9182 ± 0.0010	+0.0045	0.7579	0.9102 ± 0.0044	+0.0026	1.0000

Values are reported as mean ± standard deviation across five identical outer-test folds. Δ values denote the paired mean difference calculated as full MFMNet minus the corresponding ablated variant. Two-sided paired t-tests were performed on identical folds, with Holm correction applied separately to the five primary AUROC comparisons and the five primary PR-AUC comparisons. None of the adjusted comparisons reached the 0.05 significance threshold. GCN-only, GIN-only, and GAT-only models were evaluated separately as descriptive branch-complementarity diagnostics and were not included in the primary Holm correction families.

### Computational efficiency and scalability

The full MFMNet backbone contained approximately 2.65 million trainable parameters. Across the five outer folds, the mean active training time was approximately 27.6 min per fold, and the mean peak accelerator memory usage during training was approximately 565.3 MB. At an inference batch size of 64, the mean inference time was approximately 1.00 ms per sample, corresponding to a throughput of approximately 1,003 samples/s. When the inference batch size was increased from 32 to 512, throughput increased from approximately 804–1,256 samples/s, while peak accelerator memory usage increased from approximately 123 MB to 1.05 GB. These measurements characterize the computational requirements of MFMNet under the evaluated hardware and software configuration and illustrate the trade-off between inference throughput and memory consumption as batch size increases.

### Independent validation on the AstraZeneca dataset

To further evaluate MFMNet beyond the five-fold cross-validation analysis, the model was evaluated on the independent AstraZeneca dataset [39]. MFMNet achieved an accuracy of 93.3% on this independent test set. This result provides an additional independent-set assessment of model performance outside the five-fold cross-validation partitions.

### Additional cross-dataset external evaluation

Beyond the AstraZeneca independent test set, the embedding-independent MFMNet configuration was further evaluated on the FRIEDMAN and NCI-ALMANAC datasets. On FRIEDMAN, the model achieved an AUROC of 0.7704 and a PR-AUC of 0.6602, with 95% confidence intervals of 0.5031–0.9500 and 0.2544–0.9028, respectively. On NCI-ALMANAC, the corresponding AUROC and PR-AUC were 0.5407 and 0.2455, with 95% confidence intervals of 0.4817–0.5975 and 0.1928–0.3053, respectively. The FRIEDMAN results indicated retained cross-study predictive ability, whereas performance decreased substantially on NCI-ALMANAC, showing that external performance varied across datasets.

## Discussion

This study introduces MFMNet, a computational framework developed to predict synergistic drug combinations in cancer cell lines by integrating complementary molecular graph representations with drug embeddings and cell-line transcriptomic features. In the initial five-fold cross-validation analysis, MFMNet achieved a mean five-fold AUROC of 0.9301 ± 0.0036. Under the stricter fold-matched evaluation protocol, in which model selection was performed exclusively using an inner validation subset and each outer-test fold remained isolated until checkpoint selection was completed, MFMNet achieved an AUROC of 0.9227 ± 0.0055 and a PR-AUC of 0.9127 ± 0.0084. Fold-matched comparisons showed statistically significant improvements in both AUROC and PR-AUC over DeepDDS-GCN, DeepDDS-GAT, and XGBoost after Holm correction. The improvements over the two DeepDDS variants were larger, whereas the differences relative to XGBoost were modest in magnitude. Dropout, L2 regularization, validation-based early stopping, and a consistent hyperparameter configuration across the five outer folds were used to reduce the risk of overfitting. The relatively small variation in AUROC across the outer-test folds further indicates stable performance across the evaluated record-level data partitions.

Recent graph- and attention-based approaches, including DeepDDS [23], AttenSyn [24], HANSynergy [25], and SynergyX [26], have improved drug-synergy prediction through molecular graph learning, heterogeneous biological modeling, or cross-modal attention. In contrast, MFMNet integrates heterogeneous GCN, GIN, and GAT representations through TGP and further coordinates these representations using CSAR. Although MFMNet showed significant improvements over the fold-matched DeepDDS variants in the present study, direct performance comparisons with other recent models remain limited by differences in datasets and evaluation protocols. DeepDDS and AttenSyn emphasize single graph representations or drug-pair attention, whereas HANSynergy and SynergyX incorporate richer heterogeneous or multi-omic information; MFMNet instead focuses on complementary GCN/GIN/GAT representations and adaptive fusion. The reduced performance on NCI-ALMANAC further indicates that this architectural advantage does not necessarily translate into robust cross-dataset generalization.

Independent validation on the AstraZeneca test set yielded an accuracy of 93.3%. Additional evaluation on the FRIEDMAN and NCI-ALMANAC datasets provided a broader assessment of cross-dataset transferability. The embedding-independent MFMNet configuration achieved an AUROC of 0.7704 and a PR-AUC of 0.6602 on the FRIEDMAN dataset, providing supportive evidence of cross-study transferability, although the small number of classifiable records limits the precision of this estimate. In contrast, performance decreased substantially on NCI-ALMANAC, with an AUROC of 0.5407 and a PR-AUC of 0.2455. The difference between the two external evaluations indicates that model performance can vary considerably across datasets and may be affected by differences in experimental source, dataset composition, and synergy-score definition.

Among the top-ranked predictions, no direct published evidence for the exact drug pair was identified for VINBLASTINE–L778123, MITOMYCINC–DASATINIB, TOPOTECAN–BEZ-235, and L778123–ERLOTINIB. The absence of direct reports does not establish novelty, but the known pharmacological properties of the individual agents provide biological context for these predictions. VINBLASTINE suppresses microtubule dynamics and interferes with mitotic progression [40], whereas L-778,123 inhibits farnesyl:protein transferase and geranylgeranyl: protein transferase type I and thereby interferes with protein prenylation [41]. These distinct effects suggest a possible complementary interaction between disruption of mitotic dynamics and prenylation-dependent signaling. For MITOMYCINC–DASATINIB, mitomycin-mediated DNA damage provides genotoxic stress [42,43], whereas dasatinib-mediated Src inhibition may attenuate signaling associated with cell survival and the DNA-damage response. Dasatinib has also been reported to enhance the cytotoxicity of cisplatin in several non-small-cell lung cancer cell lines and to suppress cisplatin-induced expression of DNA-repair and DNA-synthesis genes [44], providing pathway-level context for this prediction. For TOPOTECAN–BEZ-235, topotecan-induced cytotoxicity has been associated with suppression of the PI3K–Akt survival pathway, and constitutive Akt activation has been reported to reduce cellular sensitivity to topotecan [45]. BEZ235 inhibits PI3K/mTOR signaling and has also been shown to inhibit ATM- and DNA-PK-dependent repair of DNA double-strand breaks [46]. These observations provide a plausible basis for combined suppression of prosurvival signaling and DNA-damage repair. Finally, L-778,123 inhibits protein prenyltransferases involved in the processing of multiple signaling proteins [41], whereas erlotinib inhibits EGFR signaling [47]. Although direct evidence for L778123–ERLOTINIB was not identified, a phase I study of the related farnesyltransferase inhibitor tipifarnib combined with erlotinib was initiated on the basis of preclinical cytotoxic synergy between those agents [47], providing indirect class-level context for the predicted combination. These observations provide testable hypotheses for subsequent biological investigation rather than experimental confirmation of the predicted drug combinations.

Several limitations should be considered when interpreting the present results. Although the dataset contains 13,243 labeled drug-combination records, it includes only 38 unique drugs and 31 cell lines, which limits the chemical and cellular diversity represented in the current analysis. In addition, the record-level five-fold evaluation does not specifically assess generalization to entirely unseen drugs or unseen cell lines, and the additional FRIEDMAN and NCI-ALMANAC evaluations were restricted to drugs and cell lines for which transferable model inputs were available. The reduced performance observed on NCI-ALMANAC further indicates that cross-dataset differences can affect predictive performance. MFMNet currently integrates molecular graphs, drug embeddings, and cell-line transcriptomic profiles, without additional information such as drug-metabolism characteristics, three-dimensional molecular structures, or broader multi-omics features. Future studies should therefore evaluate MFMNet using broader drug and cell-line collections, including unseen-drug and unseen-cell-line settings, and investigate whether additional molecular and cellular information further improves generalization. Experimental evaluation of the four predicted combinations lacking direct literature support would also be valuable for assessing their biological relevance. Overall, the present results support MFMNet as a computational framework for prioritizing candidate synergistic drug combinations, while broader computational and experimental validation remains necessary to establish its generalizability.

The controlled ablation analysis showed that the three TGP branches contributed differently to overall performance, while their low representation similarities and distinct prediction errors indicated that they captured complementary molecular information. CSAR also showed a modest descriptive advantage over mean fusion and simple concatenation, although the differences were not statistically significant after Holm correction. These results support the use of TGP and CSAR as complementary representation and adaptive fusion mechanisms rather than as components that independently guarantee significant performance gains.

## Conclusion

In this study, we developed MFMNet for synergistic drug-pair prediction by integrating a Tri-Layer Graph Pyramid (TGP) with a Cross-Scale Attention Router (CSAR). The TGP combines molecular representations generated by GCN, GIN, and GAT branches, while CSAR adaptively integrates the heterogeneous graph features with drug embeddings and cell-line transcriptomic information. Under the uniform fold-matched protocol, MFMNet achieved the highest mean AUROC and PR-AUC among the evaluated principal baseline models. Controlled ablation and branch-level diagnostic analyses indicated that the heterogeneous graph branches learned partially complementary and non-redundant representations, while CSAR showed a descriptive advantage over mean fusion and simple concatenation, although the ablation differences were not statistically significant after Holm correction. Additional cross-dataset analyses showed supportive transferability on the FRIEDMAN dataset but reduced performance on NCI-ALMANAC, indicating that predictive performance remains sensitive to dataset differences. These findings support the use of MFMNet for computational prioritization of candidate synergistic drug combinations. Further evaluation on broader drug and cell-line collections, together with experimental validation of selected predictions, will be important for assessing its generalizability and biological relevance. Because all performance estimates were obtained by record-level cross-validation within a single dataset, generalization to entirely unseen drugs and cell lines remains to be established in future work.
