# Supplementary material for: MFMNet: Adaptive tri-layer graph pyramid with cross-scale attention router for synergistic drug prediction
Source: PLoS One. 2026 Nov 10;21(11):e0362586. doi: 10.1371/journal.pone.0362586 (PMC13750550; doi:10.1371/journal.pone.0362586)
Supplement: Supplementary file 1 [file pone.0362586.s002.doc]

| School of Medicine and Information Engineering, Anhui University of Chinese Medicine, Hefei, Anhui, 230012, China |  | Dr. Li Jin  Associate Professor, Master's Supervisor  School of Medicine and Information Engineering  Anhui University of Chinese Medicine  Email: jinli@ahtcm.edu.cn |
| --- | --- | --- |

**Comments to the reviewers and all changes with the draft are summarized below**

**Journal Requirements**

1. PLOS ONE style and file-naming requirements

Comment: Please ensure that your manuscript meets PLOS ONE's style requirements, including those for file naming.

**Author response:** Thank you for this guidance. We revised the title to sentence case, added a short title, removed the postal code from the affiliation, standardized the corresponding-author information, and revised the section headings, figure citations, figure captions, and manuscript formatting to follow the PLOS ONE style requirements. We also removed the standalone Competing interests section from the manuscript because this information is provided through the PLOS submission system; the authors’ declaration that no competing interests exist is retained in the Cover Letter. We will also ensure that the final figure files are named and uploaded in accordance with the journal’s file-naming requirements.

**Location in the revised manuscript:** Title page, lines 1–10; figure legends, lines 155–157, 433–434, 458–462, 477–479, and 525–529; References, lines 764–938.

2. Code-sharing requirement

Comment: Author-generated code underpinning the findings should be made available without restrictions and in a form that facilitates reproducibility and reuse.

**Author response:** Thank you for highlighting the code-sharing requirement. The MFMNet code is publicly available in the project repository. For this revision, the repository has been updated to include the final scripts used for the analyses reported in the revised manuscript, including the fold-matched statistical comparisons, ablation experiments, and computational-resource measurements, together with the corresponding outputs and information on the software environment and execution procedures. These materials are provided to facilitate reproducibility and reuse of the reported analyses.

**Location in the revised manuscript:** Data availability, lines 758–763.

3. Funding disclosure and Role of the Funders

Comment: Please state what role the funders took in the study. If the funders had no role, include the journal-provided statement in the cover letter.

**Author response:** Thank you for this instruction. We have revised the Funding Statement to reflect the current funding information. The revised statement includes the Provincial Teaching Research Key Project of Anhui Province (Li Jin, Grant No. 2024jyxm0215), the Youth Project of Anhui Provincial Department of Education (Jingwen Pan, Grant No. 2025AHGXZK40314), and the Teaching Research Key Project of Anhui Provincial Department of Education (Liping Sun, Grant No. 2025jyxm0137). The previously listed funding sources that are no longer applicable have been removed. We have also clarified the role of the funders in the revised Cover Letter. The funders provided financial support for the research activities and publication-related expenses but had no role in study design, data collection and analysis, decision to publish, or preparation of the manuscript.

**Location in the revised submission:** Cover Letter, Funding Statement and Role of the Funders paragraphs on page 1.

4. Remove funding information from Acknowledgments

Comment: Funding information should not appear in the Acknowledgments section or other areas of the manuscript; please remove it and provide the amended statement in the cover letter.

**Author response:** Thank you for this instruction. We have removed all funding-related information from the Acknowledgments section of the revised manuscript. Funding information is therefore no longer duplicated in the manuscript. The amended Funding Statement and the Role of the Funders statement are provided in the revised Cover Letter for updating the corresponding information in the submission system.

**Location in the revised submission:** Funding information is provided only in the Cover Letter, page 1; no funding information or Acknowledgments section containing funding information remains in the revised manuscript.

5. Ethics statement location

Comment: The ethics statement should appear only in the Methods section and should be removed from any other section.

**Author response:** Thank you for this guidance. The revised manuscript now contains the ethics statement only in the Materials and methods section. The separate “Ethical standards” section that appeared outside the Methods section has been removed to avoid duplication and to comply with the journal’s requirement.

**Location in the revised manuscript:** Materials and methods, “Ethics statement,” lines 184–191.

Reviewer #1

Major Comment 1 — Statistical significance testing

Comment: While Table 2 reports performance metrics with standard deviations, formal statistical significance testing is missing. Appropriate paired tests and p-values should be provided for comparisons with baseline models such as DeepDDS and XGBoost.

**Author response:** We agree that formal statistical testing is necessary. In the revised manuscript, we performed fold-matched comparisons between MFMNet and DeepDDS-GCN, DeepDDS-GAT, and XGBoost using identical outer-test fold assignments. Two-sided paired t-tests were applied to AUROC and PR-AUC values across matched folds, and Holm correction was performed separately within the AUROC and PR-AUC comparison families. Table 3 reports the mean paired differences, 95% confidence intervals, raw paired p-values, and Holm-adjusted p-values. All six fold-matched comparisons remained statistically significant after Holm correction (adjusted p < 0.05). We also clarified that Table 2 summarizes the initial five-fold cross-validation analysis, whereas Table 3 provides the formal inference under the stricter fold-matched evaluation protocol.

**Location in the revised manuscript:** “Statistical analysis,” lines 357–379; fold-matched statistical-comparison results, lines 492–504; Table 3 and its explanatory text, lines 505–510.

Major Comment 2 — Model complexity versus sample size

Comment: The reviewer requests a stronger explanation of how overfitting is controlled given the sample size and the complexity of the architecture, beyond early stopping.

**Author response:** Thank you for raising this important point. We revised both the Materials and methods and Discussion sections to clarify the architecture actually used in the reported experiments and the measures used to reduce the risk of overfitting. For each outer fold, an inner validation subset was used exclusively for model selection and early stopping, while the corresponding outer-test fold remained isolated until the best checkpoint had been selected. In addition to early stopping, the training procedure incorporated dropout, L2 regularization, learning-rate scheduling, and a fixed hyperparameter configuration across all outer folds. We also clarified that these measures reduce, but do not eliminate, the risk of overfitting, particularly because the current dataset contains a limited number of unique drugs and cell lines. Accordingly, the Discussion now distinguishes stability across the evaluated record-level folds from generalization to entirely unseen drugs or cell lines. To make model capacity and computational cost transparent, we additionally report the number of trainable parameters, training time, inference latency and throughput, GPU memory consumption, and scalability across inference batch sizes.

**Location in the revised manuscript:** “Hyperparameter settings,” lines 211–234; “Computational efficiency and scalability,” lines 568–582; Discussion, lines 604–626 and 693–718.

Major Comment 3 — Biological plausibility of TOPOTECAN + BEZ-235 and L778123 + ERLOTINIB

Comment: The reviewer asks for a discussion of biological plausibility or potential mechanisms for the two novel combinations that lack direct literature validation.

**Author response:** We appreciate this important suggestion. During revision, we re-evaluated the literature support for all ten top-ranked drug combinations in Table 1. This re-evaluation identified four combinations, rather than two, for which no direct published evidence of the exact drug pair was found: VINBLASTINE–L778123, MITOMYCINE–DASATINIB, TOPOTECAN–BEZ-235, and L778123–ERLOTINIB. We have therefore corrected Table 1 accordingly and added a focused discussion of the biological plausibility of all four combinations, with particular attention to the two combinations highlighted by the reviewer. The discussion is based on the known pharmacological actions and relevant pathway-level evidence for the individual agents and related therapeutic strategies. For TOPOTECAN–BEZ-235, we discuss the potential complementarity between topotecan-associated DNA damage and PI3K–Akt signaling suppression and the inhibition of PI3K/mTOR and DNA-damage repair by BEZ235. For L778123–ERLOTINIB, we discuss the potential complementary effects of prenyltransferase inhibition and EGFR pathway inhibition, together with indirect class-level evidence from related therapeutic strategies. We explicitly distinguish these mechanistic considerations from direct experimental validation and clarify that dedicated in vitro studies are required to determine whether these predicted combinations are genuinely synergistic.

**Location in the revised manuscript:** Results, lines 406–418 and Table 1 at line 420; Discussion, lines 656–692.

Minor Comment 1 — Typographical and translation errors

Comment: The reviewer identifies “adaptive Fri-layer graph pyramid,” “Caesar's dynamic weight assignment,” and “ethi cal” as errors.

**Author response:** Thank you for identifying these errors. We have corrected “adaptive Fri-layer graph pyramid” to “adaptive Tri-Layer Graph Pyramid,” replaced the erroneous “Caesar's” wording with the correct Cross-Scale Attention Router (CSAR) terminology, and removed the former standalone “Ethical standards” section outside the Methods section. The ethics statement is now presented only in the Materials and methods section, in accordance with the journal’s requirement. We also reviewed the manuscript for related typographical and terminology inconsistencies and standardized the terminology for the Tri-Layer Graph Pyramid (TGP) and Cross-Scale Attention Router (CSAR) throughout the revised manuscript.

**Location in the revised manuscript:** Introduction, lines 137–153; “Ethics statement,” lines 184–191; TGP/GCN/GIN/GAT/CSAR methodological descriptions, lines 236–343; Conclusion, lines 726–752.

Minor Comment 2 — Informal tone

Comment: The reviewer notes informal phrases such as “They don't block,” “And they've also cut,” and “What's worse.”

**Author response:** Thank you for this comment. We have revised the informal and conversational expressions throughout the manuscript to use more precise scientific language. In the Introduction, “They don't block...” was replaced with a statement explaining that single-agent therapies may fail to suppress redundant signaling pathways; “And they've also cut side effects” was revised to describe, more cautiously, the circumstances under which combination strategies may reduce treatment-related adverse effects; and “What's worse” was replaced with “Moreover.” We also revised other conversational expressions in the Results and Discussion sections to maintain a consistent formal academic style throughout the manuscript. In addition, statements regarding model superiority, biological interpretation, and translational relevance were revised where necessary to avoid overly strong or unsupported wording.

**Location in the revised manuscript:** Introduction, lines 40–74; Results, lines 406–418 and 492–504; Discussion, lines 603–725.

Minor Comment 3 — Syntactical clarity

Comment: The reviewer highlights the sentence “In particular, layers from the l to the l+2 layer add some features...” as ambiguous.

**Author response:** Thank you for pointing this out. We agree that the sentence was ambiguous. The sentence has been removed from the revised manuscript and replaced by a detailed description of the actual implemented architecture and information flow. The revised Materials and methods section now describes the GCN, GIN, and graph-attention branches separately, including their layer dimensions and processing sequence. The graph-attention branch is explicitly described as being implemented using the GATv2Conv operator. We also describe how the three 128-dimensional branch outputs are concatenated into a 384-dimensional node representation, adaptively reweighted by the Cross-Scale Attention Router, projected to 128 dimensions, and subsequently subjected to masked mean pooling over valid atomic positions before multimodal classification. These revisions ensure that the methodological description corresponds to the implemented model rather than relying on the previous ambiguous layer-to-layer wording.

**Location in the revised manuscript:** “GCN,” “GIN,” and “GAT,” lines 236–294; “Adaptive feature fusion module,” lines 318–343; “Classification module,” lines 344–355.

**Reviewer #2**

Comment 1 — Novelty relative to DeepDDS and recent graph-attention models

Comment: The novelty of MFMNet should be more clearly distinguished from existing GNN-based drug-synergy models, particularly DeepDDS and recent graph-attention architectures.

**Author response:** Thank you for this important suggestion. We have revised the Introduction to more clearly position MFMNet relative to DeepDDS and more recent graph- and attention-based drug-synergy prediction models. Specifically, we added a focused comparison with DeepDDS and representative recent approaches using graph attention, heterogeneous graph attention, drug-pair attention, and cross-modal attention. The revised discussion clarifies that the contribution of MFMNet does not lie simply in introducing an attention mechanism. Instead, MFMNet combines complementary molecular representations generated by graph convolutional, graph isomorphism, and graph-attention operations within the Tri-Layer Graph Pyramid (TGP), and uses the Cross-Scale Attention Router (CSAR) to coordinate information across these representations before integration with cell-line features. We have therefore clarified that the principal architectural distinction of MFMNet is its coordinated multi-scale graph representation and cross-scale routing strategy.

**Location in the revised manuscript:** Introduction, lines 118–153.

Comment 2 — Additional independent external validation

Comment: Validation on additional publicly available drug-synergy datasets beyond the reported AstraZeneca test set is recommended.

**Author response:** We thank the reviewer for this important suggestion. We added cross-dataset evaluations using two publicly available drug-combination datasets beyond the model-development data. To avoid selecting an external dataset on the basis of model performance, candidate DrugComb studies were screened before inference according to predefined criteria, including explicit study provenance, compatibility with the available molecular-graph and cell-line input features, removal of exact O’Neil drug-pair–cell-line overlaps, and availability of both outcome classes under the fixed Loewe-based classification rule. The source-resolved FRIEDMAN study satisfied these criteria. After removal of exact O’Neil overlaps and exclusion of records with Loewe scores between 0 and 10, 39 external records remained, comprising 9 synergistic and 30 non-synergistic combinations across 10 drugs and 5 cell lines.

During preparation of the external evaluation, we found that the supplied 768-dimensional auxiliary drug embeddings were record-dependent and could not be reconstructed consistently for independent drug-pair–cell-line records. Arbitrary external imputation of these features was therefore avoided. The additional evaluations used the pre-defined embedding-independent MFMNet configuration, which retained the GCN, GIN, and GATv2 molecular-graph branches, the Cross-Scale Attention Router, the cell-line encoder, and the downstream classifier. Five checkpoints selected exclusively using the O’Neil training and validation data were frozen before external inference and combined using fixed equal weights. No external labels were used for checkpoint selection, threshold tuning, feature construction, or ensemble weighting.

On the FRIEDMAN external study, this configuration achieved an AUROC of 0.7704 and a PR-AUC of 0.6602, with bootstrap 95% confidence intervals of 0.5031–0.9500 and 0.2544–0.9028, respectively. Because only 39 classifiable external records were available, we interpret this result cautiously as supportive evidence of cross-study transferability rather than definitive evidence of broad generalization.

We additionally evaluated the same frozen configuration on 864 NCI-ALMANAC records after removal of exact O’Neil drug-pair–cell-line overlaps. The resulting AUROC was 0.5407 and the PR-AUC was 0.2455, with 95% confidence intervals of 0.4817–0.5975 and 0.1928–0.3053, respectively. We retained this less favorable external result in the revised analysis because it provides important information regarding cross-dataset distribution and endpoint shift. In particular, NCI-ALMANAC ComboScore and the O’Neil Loewe score are not identical response measures. The revised manuscript now reports both the supportive FRIEDMAN result and the more challenging NCI-ALMANAC result and explicitly limits the corresponding generalization claims.

**Location in the revised manuscript:** “Additional external validation,” lines 380–401; “Additional cross-dataset external evaluation,” lines 591–602; Discussion, lines 643–655 and 693–718.

Comment 3 — More detailed TGP and CSAR architecture/information flow

Comment: The architectural design and information flow of the Tri-Layer Graph Pyramid and Cross-Scale Attention Router should be explained more clearly for reproducibility.

**Author response:** Thank you for this comment. We substantially expanded the Materials and methods section to provide a stepwise description of the implemented TGP and CSAR architecture and information flow. The revised manuscript separately describes the GCN, GIN, and graph-attention branches, including their layer dimensions and processing sequences. The graph-attention branch is explicitly described as being implemented using the GATv2Conv operator. The 128-dimensional node representations produced by the three graph branches are concatenated into a 384-dimensional representation, which is adaptively reweighted by the CSAR, projected to 128 dimensions, and subjected to masked mean pooling over valid atomic positions. The resulting graph representations are then integrated with the two drug embeddings and the cell-line transcriptomic representation to form the input to the final classifier. These revisions align the methodological description with the implemented architecture and provide a clearer account of the complete information flow for reproducibility.

**Location in the revised manuscript:** “GCN,” “GIN,” and “GAT,” lines 236–294; “Adaptive feature fusion module,” lines 318–343; “Classification module,” lines 344–355.

Comment 4 — Biological interpretation and pathway-level validation

Comment: Additional mechanistic analysis and pathway-level validation would strengthen the biological significance of the predicted synergistic drug pairs.

**Author response:** We agree that stronger biological interpretation improves the biological significance of the predicted combinations. Because no direct pathway-level experiments were performed in the present study, we did not present the proposed mechanisms as experimentally validated pathways. Instead, we added a literature-supported mechanistic discussion of the top-ranked combinations for which no direct evidence of the exact drug pair was identified. In particular, the revised Discussion examines plausible biological interactions for VINBLASTINE–L778123, MITOMYCINE–DASATINIB, TOPOTECAN–BEZ-235, and L778123–ERLOTINIB on the basis of the established pharmacological actions of the individual agents and relevant pathway-level evidence. For TOPOTECAN–BEZ-235, we discuss the potential complementarity between topotecan-associated DNA damage and PI3K–Akt signaling suppression and the PI3K/mTOR and DNA-repair inhibitory effects of BEZ235. For L778123–ERLOTINIB, we discuss the potential complementarity between prenyltransferase inhibition and EGFR pathway inhibition. We explicitly characterize these explanations as testable mechanistic hypotheses rather than direct pathway-level validation and state that dedicated biological experiments will be required for confirmation.

**Location in the revised manuscript:** Results, lines 406–418 and Table 1 at line 420; Discussion, lines 656–692.

Comment 5 — Statistical significance and confidence intervals

Comment: The reviewer requests statistical significance testing and confidence intervals for comparisons with competing methods.

**Author response:** Thank you for this important point. We added a dedicated statistical-analysis subsection and performed fold-matched comparisons using identical outer-test fold assignments. MFMNet was compared with DeepDDS-GCN, DeepDDS-GAT, and XGBoost for AUROC and PR-AUC using two-sided paired t-tests. The revised manuscript reports mean paired differences, 95% confidence intervals, raw paired p-values, and Holm-adjusted p-values in Table 3, with Holm correction applied separately to the AUROC and PR-AUC comparison families. All six fold-matched comparisons remained statistically significant after correction (adjusted p < 0.05). We also distinguish the descriptive initial five-fold cross-validation results in Table 2 from the stricter fold-matched statistical comparison in Table 3.

**Location in the revised manuscript:** “Statistical analysis,” lines 357–379; fold-matched statistical-comparison results, lines 492–504; Table 3 and its explanatory text, lines 505–510.

Comment 6 — Experimental validation of newly predicted combinations

Comment: The reviewer states that experimental validation of newly predicted drug combinations is necessary to demonstrate translational potential.

**Author response:** We appreciate the reviewer's emphasis on experimental validation and agree that wet-lab validation is important for establishing the translational relevance of newly predicted drug combinations. The present study is computational in scope, and no new in vitro experiments were performed in this revision. We have therefore revised the manuscript to avoid presenting the newly predicted combinations as experimentally validated synergies. The Results and Discussion now distinguish literature-supported mechanistic hypotheses from direct biological validation, and the manuscript explicitly states that dedicated in vitro studies will be required to determine whether these predicted interactions are genuinely synergistic. We also moderated statements concerning clinical and translational potential in the Abstract, Discussion, and Conclusion and explicitly identified the absence of wet-lab validation as a limitation of the current study. Accordingly, the predicted combinations are presented as candidates for subsequent experimental investigation rather than as experimentally confirmed therapeutic combinations.

**Location in the revised manuscript:** Abstract, lines 12–36; Results, lines 406–418 and Table 1 at line 420; Discussion, lines 656–718; Conclusion, lines 726–752.

Comment 7 — Comprehensive ablation studies

Comment: The reviewer requests quantitative evaluation of the contributions of the Tri-Layer Graph Pyramid, Cross-Scale Attention Router, and feature-fusion strategy.

**Author response:** Thank you for this suggestion. We have added a controlled ablation analysis to quantitatively evaluate the contributions of the Tri-Layer Graph Pyramid (TGP), Cross-Scale Attention Router (CSAR), and graph-feature fusion strategy. All variants were evaluated using the same five outer-test folds, inner-validation procedure, optimization budget, stopping rule, and controlled fold-specific initialization.

Specifically, we compared the full MFMNet with variants removing the GCN, GIN, or GAT branch, as well as variants replacing CSAR with unweighted mean fusion or simple concatenation followed by linear projection. The full model achieved an AUROC of 0.9227 ± 0.0055 and a PR-AUC of 0.9127 ± 0.0084. Removing GCN and GIN resulted in mean AUROCs of 0.9213 ± 0.0039 and 0.9206 ± 0.0024, respectively, whereas removing GAT yielded a similar AUROC of 0.9229 ± 0.0032. Replacing CSAR with mean fusion reduced the mean AUROC to 0.9192 ± 0.0041, while simple concatenation yielded 0.9182 ± 0.0010. The corresponding mean AUROC differences in favor of the full model were 0.0035 and 0.0045 for mean fusion and simple concatenation, respectively. Paired comparisons were performed across identical folds with Holm correction; none of the primary ablation comparisons reached statistical significance after multiple-comparison correction.

We further added branch-level diagnostic analyses to examine complementarity among the GCN, GIN, and GAT branches. Pairwise prediction disagreement rates were approximately 8.7%–9.1%, each branch uniquely classified a subset of samples correctly, and pairwise representation similarities were low overall. These findings indicate that the three graph encoders capture substantially non-redundant and partially complementary molecular information, although their marginal contributions to aggregate predictive performance are heterogeneous. We have revised the manuscript accordingly and have avoided interpreting the non-significant ablation differences as statistically significant improvements.

**Location in the revised manuscript:** “Statistical analysis,” lines 366–379; “Controlled ablation analysis of TGP and CSAR,” lines 530–567; Discussion, lines 719–725.

Comment 8 — Computational complexity, time, memory, and scalability

Comment: The reviewer requests computational complexity, training time, memory consumption, and scalability information.

**Author response:** Thank you for this comment. We added a dedicated computational-efficiency and scalability analysis using the revised model implementation. The full MFMNet backbone contained approximately 2.65 million trainable parameters. Across the five outer folds, the mean active training time was approximately 27.6 min per fold and the mean peak accelerator memory usage during training was approximately 565.3 MB. At an inference batch size of 64, the mean inference time was approximately 1.00 ms per sample, corresponding to a throughput of approximately 1,003 samples/s. When the inference batch size was increased from 32 to 512, throughput increased from approximately 804 to 1,256 samples/s, while peak accelerator memory usage increased from approximately 123 MB to 1.05 GB. These results were added to make the computational requirements and scalability of MFMNet more transparent.

**Location in the revised manuscript:** “Computational efficiency and scalability,” lines 568–582.

Comment 9 — Reproducibility details

Comment: More detail is requested for data preprocessing, class-imbalance handling, negative-sample generation, and hyperparameter optimization.

**Author response:** We agree and have expanded the Materials and methods section to provide a more explicit account of the data-processing and training procedures. The revised manuscript now reports the dataset size, numbers of unique drugs and cell lines, synergy-score criteria used for binary-label construction, class distribution, and molecular-graph preprocessing procedure. We clarify that no additional negative samples were synthetically generated and that the original class distribution was retained without oversampling, undersampling, or class-weighted loss. We also describe the five-fold outer-test partitioning, inner validation subsets used for model selection, fixed random-seed strategy, optimization settings, learning-rate scheduling, regularization, and early-stopping procedure. The same hyperparameter configuration was maintained across all outer folds, and no fold-specific hyperparameter tuning was performed using the corresponding outer-test data. The accompanying public repository also provides the software and execution information used for the revised experiments to facilitate reproducibility.

**Location in the revised manuscript:** “Data source and label definition,” lines 159–182; “Data preprocessing,” lines 193–209; “Hyperparameter settings,” lines 211–234.

Comment 10 — Figure quality and AI-assisted figure generation

Comment: Several figures appear highly stylized and schematic; figure quality should be improved, and AI-assisted illustration generation should be disclosed if applicable.

**Author response:** Thank you for this comment. We revised the figures to improve scientific presentation and readability by simplifying schematic elements, standardizing fonts, labels, line styles, and panel formatting, and regenerating quantitative plots from the verified experimental results. Figure 1 was reconstructed as an editable schematic so that the TGP, CSAR, masked pooling, multimodal integration, and classifier information flow correspond directly to the implemented architecture. The quantitative performance figures were also updated to match the final reported results. AI-assisted tools were used during preliminary figure drafting, and Grammarly was used solely for language proofreading. The final figures were reconstructed and verified by the authors against the implemented model and experimental results.

**Location in the revised manuscript:** Fig 1 legend, lines 155–157; Fig 2 legend, lines 433–434; Fig 3 legend, lines 458–462; Fig 4 legend, lines 477–479; Fig 5 legend, lines 525–529.

Comment 11 — Critical comparison with recent state-of-the-art methods

Comment: The Discussion should include a more critical comparison with recent state-of-the-art deep-learning methods and discuss the limitations of MFMNet.

**Author response:** Thank you for this suggestion. We have revised the Discussion to include a concise comparison with recent graph- and attention-based drug-synergy prediction methods, including DeepDDS, AttenSyn, HANSynergy, and SynergyX. The revised text highlights the distinction of MFMNet in jointly integrating heterogeneous GCN, GIN, and GAT representations through TGP and coordinating these representations using CSAR. We also clarify that, although MFMNet showed statistically significant improvements over the fold-matched DeepDDS variants in the present study, direct numerical comparisons with other recent methods should be interpreted cautiously because of differences in datasets and evaluation protocols. The limitations of MFMNet are further discussed separately in the revised Discussion.

**Location in the revised manuscript:** Discussion, lines 627–642.

Comment 12 — Limitations and generalization to unseen drugs/cell lines

Comment: The reviewer requests a fuller limitations discussion, including the relatively small dataset, limited biological validation, and challenges in generalizing to unseen drugs and cancer cell lines.

**Author response:** We agree and have substantially expanded the limitations discussion. The revised manuscript now explicitly states that, although the analyzed dataset contains 13,243 labeled drug-combination records, it includes only 38 unique drugs and 31 cancer cell lines, thereby limiting the chemical and cellular diversity represented in the study. We further clarify that the current five-fold record-level evaluation does not specifically assess generalization to entirely unseen drugs or unseen cell lines and that stability across the evaluated outer folds should not be interpreted as evidence of out-of-distribution generalization. The revised Discussion also identifies the absence of direct pathway-level experimental validation and wet-lab validation of the newly predicted combinations as important limitations. In addition, we note that the current model primarily uses molecular graphs, drug embeddings, and cell-line transcriptomic features without broader multi-omics or additional drug-metabolism information. Translational claims have therefore been moderated, and validation on broader datasets and under unseen-drug and unseen-cell-line settings is identified as an important direction for future work.

**Location in the revised manuscript:** Discussion, lines 643–655 and 693–718.
