# Supplementary material for: MFMNet: Adaptive tri-layer graph pyramid with cross-scale attention router for synergistic drug prediction
Source: PLoS One. 2026 Nov 10;21(11):e0362586. doi: 10.1371/journal.pone.0362586 (PMC13750550; doi:10.1371/journal.pone.0362586)
Supplement: Supplementary file 2 [file pone.0362586.s003.doc]

| School of Medicine and Information Engineering, Anhui University of Chinese Medicine, Hefei, Anhui, 230012, China |  | Dr. Li Jin  Associate Professor, Master's Supervisor  School of Medicine and Information Engineering  Anhui University of Chinese Medicine  Email: jinli@ahtcm.edu.cn |
| --- | --- | --- |

**Comments to the reviewers and all changes with the draft are summarized below**

Reviewer #1

“The research is technically sound and addresses a significant bottleneck in oncology drug discovery. However, a few areas regarding statistical clarity and textual presentation require minor revisions before publication.”

**Major Comment 1 — Cross-validation and data leakage**

**Comment:** The authors appropriately utilize 5-fold cross-validation to evaluate model performance, reporting an average AUC of 0.929. To ensure absolute statistical rigor, the authors should explicitly confirm in the Methods section that hyperparameter tuning (mentioned in section 2.3) was conducted strictly within the training folds (e.g., using nested cross-validation) and that the test folds remained entirely unseen to prevent data leakage.

**Author response:** We agree, and an explicit statement has been added at the end of the “Hyperparameter settings” section: “Hyperparameter tuning and model selection were therefore performed exclusively on the training and validation data of each outer fold, and no information from the outer-test folds—including records, labels, or summary statistics—was used at any stage of model development; each outer-test fold was evaluated exactly once, after the final checkpoint had been frozen.” The cross-validation architecture—an outer five-fold partition with a stratified inner-validation split, early stopping on validation AUROC only, and post hoc evaluation of the untouched outer-test fold—corresponds exactly to the protocol the reviewer describes, and the new sentence states these guarantees explicitly in the Methods.

**Location in the revised manuscript:** Materials and methods, “Hyperparameter settings,” lines 240–244.

**Major Comment 2 — Data Availability Statement (README for figure replication)**

**Comment:** The authors have laudably provided GitHub links for the drug-cell combination dataset, model code, training weights, and experimental scripts under the MIT License. To fully comply with open science best practices, please ensure that the repository contains an explicit README.md with step-by-step instructions on how to replicate the specific figures (e.g., Figure 2 heatmaps and Figure 3 scatter plots) presented in the manuscript.

**Author response:** The repository now contains a README.md with (i) environment installation instructions, (ii) download links for the drug–cell-line combination dataset, and (iii) step-by-step commands and scripts for reproducing the manuscript's data-driven figures: Figure 2 (atomic-feature similarity heatmaps) is regenerated directly from the released fold checkpoints; Figures 3–5 are regenerated from the released fold-level metric and per-sample prediction CSVs, with the commands and the file sources of every reported value documented in the README; and Figure 1 is a schematic architecture diagram and is annotated as such. The README and the accompanying scripts, per-fold metrics, per-sample predictions, and frozen checkpoints were added to the repository in this revision: https://github.com/wuyababy/MFMNET-2/tree/main (commit a6441827, https://github.com/wuyababy/MFMNET-2/commit/a644182723cb6423e6c0dc52c40390d880bfd667).

**Location in the revised manuscript:** Data availability, lines 797–799.

**Major Comment 3 — Performance Metrics (significance versus DeepDDS)**

**Comment:** Table 2 provides a comprehensive multi-metric comparison featuring standard deviations (e.g., AUC 0.9301 ± 0.030 for MFMNet). It would strengthen the statistical analysis to briefly state whether the differences between MFMNet and the second-best model (DeepDDS) are statistically significant (e.g., via a paired t-test across the folds).

**Author response:** We agree that stating the DeepDDS-specific results explicitly strengthens the statistical analysis. The fold-matched paired t-tests with Holm correction are reported in Table 3 (two-sided, computed across the five identical outer-test folds, with correction applied separately within the AUROC and PR-AUC comparison families). To make the key result explicit, the following sentence has been added immediately after the existing significance statement in the Results section: “In particular, MFMNet significantly outperformed both DeepDDS variants on both metrics under Holm-adjusted paired t-tests: p = 0.0099 (AUROC) and 0.0075 (PR-AUC) versus DeepDDS-GCN, and p = 0.0130 (AUROC) and 0.0332 (PR-AUC) versus DeepDDS-GAT.”

**Location in the revised manuscript:** Results, “Multi-metric comparative experiments using state-of-the-art methods,” lines 529–533.

**Minor Comment 1 — Language and phrasing**

**Comment:** While the manuscript is generally intelligible, the Introduction contains some colloquial or awkward phrasing that should be revised for standard academic tone. For instance, the phrase “makes single-agent treatment an automatic failure by default” is overly absolute and informal. Similarly, “Such is an age-old relationship between synergy and medicine” should be rephrased for scientific objectivity.

**Author response:** We agree that such phrasing is inappropriate for a scientific manuscript. Both sentences were already removed during the first revision and are absent from the clean manuscript; the passages noted by the reviewer correspond to the marked-up revision copy, in which they appear only as tracked deletions. The corresponding passage now reads: “Single-agent therapies may fail to suppress redundant signaling pathways; for example, the effects of EGFR inhibition in colorectal cancer can be attenuated by compensatory activation of MET signaling, contributing to treatment resistance and relapse [2].” We have re-examined the Introduction to ensure a consistent academic tone throughout.

**Location in the revised manuscript:** Introduction, lines 46–49.

**Minor Comment 2 — Typographical consistency in Table 1**

**Comment:** In Table 1, the alignment and hyphenation of drug names (e.g., “VINBLASTIN E”, “DASATINI B”) appear fragmented due to formatting. Please ensure standard nomenclature formatting in the final table

**Author response:** The drug names stored in the manuscript text are spelled correctly (e.g., VINBLASTINE, DASATINIB); the fragmentation the reviewer observed arose from column-width text wrapping in the rendered PDF, where long names were broken mid-word. In the revised manuscript we have (i) fixed the table layout (fixed-layout table with widened “Drug A” (4.32 cm) and “Drug B” (3.97 cm) columns) so that every drug name is displayed intact on a single line, and (ii) verified all drug names against standard nomenclature. During this verification we identified and corrected one genuine spelling error, “MITOMYCINE” → “MITOMYCINC”, in Table 1 and in the corresponding Results paragraph; the corrected spelling follows the drug's standard name (mitomycin C) as used in the source dataset and in reference [42].

**Location in the revised manuscript:** Table 1, line 436; Results, lines 690–700.

**Minor Comment 3 — Figure clarity (Figure 4)**

**Comment:** Figure 4 contains multiple dense histograms and ROC curves. Ensuring high-resolution vector graphics are uploaded during the revision will help readers distinguish the overlapping distributions clearly.

**Author response:** Figure 4 has been re-exported as an uncompressed high-resolution (300 dpi) TIFF with flattened RGB output and clear, uniform typography, so that the overlapping distributions can be distinguished clearly. All other figures (Figs 1–5) were re-examined and re-exported at ≥300 dpi with uniform font sizes, consistent with PLOS ONE figure specifications.

**Location in the revised manuscript:** Fig 4 legend, lines 502–504.

**Reviewer #3**

“Recommendation: Accept with Minor Revisions.” We thank the reviewer for the positive assessment and the concrete action items, which are addressed below.

**Comment 1 — Refine generalization language**

**Comment:** Review the Abstract and Conclusion to ensure all assertions strictly reflect record-level cross-validation results, explicitly noting that generalization to unseen drugs and cell lines requires further study.

**Author response:** We have revised the Abstract and Conclusion accordingly. Abstract, Conclusions (now reads): “The results support its use for computational prioritization of candidate synergistic drug combinations. All reported performance estimates refer to record-level five-fold cross-validation within the O'Neil dataset; generalization to entirely unseen drugs and cell lines requires further study.” Conclusion (added): “Because all performance estimates were obtained by record-level cross-validation within a single dataset, generalization to entirely unseen drugs and cell lines remains to be established in future work.”

**Location in the revised manuscript:** Abstract, lines 33–37; Conclusion, lines 783–785.

**Comment 2 — Code repository maintenance**

**Comment:** Ensure that execution scripts, environment specifications, and frozen checkpoint loaders for the embedding-independent model variant used in the FRIEDMAN and NCI-ALMANAC evaluations remain permanently archived in the primary GitHub repository (https://github.com/wuyababy/MFMNET-2/tree/main).

**Author response:** The execution scripts, environment specifications (requirements.txt), and the frozen checkpoints of the embedding-independent model variant (mfmnet_no_drug_embedding_fold1–5.pt) are permanently archived in the primary repository: https://github.com/wuyababy/MFMNET-2/tree/main (commit a6441827).

**Location in the revised manuscript:** Data availability, lines 797–799.

**Comment 3 — Figure text verification**

**Comment:** Verify that all reconstructed figures (Figs 1–5) maintain uniform font sizes, uncompressed high-resolution panel renderings, and clear typography in accordance with PLOS ONE final submission specifications.

**Author response:** All reconstructed figures (Figs 1–5) were re-exported with uniform font sizes, uncompressed high-resolution rendering, and clear typography in accordance with the PLOS ONE final submission specifications.

**Location in the revised manuscript:** Fig 1 legend, line 161; Fig 5 legend, lines 556–560; and the corresponding legends of Figs 2–4.
