# Supplementary material for: MFMNet: Adaptive tri-layer graph pyramid with cross-scale attention router for synergistic drug prediction
Source: PLoS One. 2026 Nov 10;21(11):e0362586. doi: 10.1371/journal.pone.0362586 (PMC13750550; doi:10.1371/journal.pone.0362586)
Supplement: Peer review history

# Peer review: Decision Letter 0

**DOI:** 10.1371/journal.pone.0362586.r001

**Authors:** Junhuang Jiang

5 Aug 2026

Dear Dr. li,

Thank you for submitting your manuscript to PLOS ONE. After careful consideration, we feel that it has merit but does not fully meet PLOS ONE’s publication criteria as it currently stands. Therefore, we invite you to submit a revised version of the manuscript that addresses the points raised during the review process.

Please submit your revised manuscript by Sep 19 2026 11:59PM. If you will need more time than this to complete your revisions, please reply to this message or contact the journal office at plosone@plos.org. When you're ready to submit your revision, log on to https://www.editorialmanager.com/pone/ and select the 'Submissions Needing Revision' folder to locate your manuscript file.

- A letter that responds to each point raised by the academic editor and reviewer(s). You should upload this letter as a separate file labeled 'Response to Reviewers'.
- A marked-up copy of your manuscript that highlights changes made to the original version. You should upload this as a separate file labeled 'Revised Manuscript with Track Changes'.
- An unmarked version of your revised paper without tracked changes. You should upload this as a separate file labeled 'Manuscript'.

If you would like to make changes to your financial disclosure, please include your updated statement in your cover letter. Guidelines for resubmitting your figure files are available below the reviewer comments at the end of this letter.

If applicable, we recommend that you deposit your laboratory protocols in protocols.io to enhance the reproducibility of your results. Protocols.io assigns your protocol its own identifier (DOI) so that it can be cited independently in the future. For instructions see: https://journals.plos.org/plosone/s/submission-guidelines#loc-laboratory-protocols. Additionally, PLOS ONE offers an option for publishing peer-reviewed Lab Protocol articles, which describe protocols hosted on protocols.io. Read more information on sharing protocols at https://plos.org/protocols?utm_medium=editorial-email&utm_source=authorletters&utm_campaign=protocols.

As the corresponding author, your ORCID iD is verified in the submission system and will appear in the published article. PLOS supports the use of ORCID, and we encourage all coauthors to register for an ORCID iD and use it as well. Please encourage your coauthors to verify their ORCID iD within the submission system before final acceptance, as unverified ORCID iDs will not appear in the published article. *Only*  the individual author can complete the verification step; PLOS staff *cannot*  verify ORCID iDs on behalf of authors.

We look forward to receiving your revised manuscript.

Kind regards,

Junhuang Jiang

Academic Editor

PLOS One

Journal requirements:

When submitting your revision, we need you to address these additional requirements.

1. Please ensure that your manuscript meets PLOS ONE's style requirements, including those for file naming. The PLOS ONE style templates can be found at

https://journals.plos.org/plosone/s/file?id=wjVg/PLOSOne_formatting_sample_main_body.pdf and

https://journals.plos.org/plosone/s/file?id=ba62/PLOSOne_formatting_sample_title_authors_affiliations.pdf

2. Please note that PLOS One has specific guidelines on code sharing for submissions in which author-generated code underpins the findings in the manuscript. In these cases, we expect all author-generated code to be made available without restrictions upon publication of the work. Please review our guidelines at https://journals.plos.org/plosone/s/materials-and-software-sharing#loc-sharing-code and ensure that your code is shared in a way that follows best practice and facilitates reproducibility and reuse.

3. Thank you for stating the following financial disclosure:

“The research is supported by the Provincial Teaching Research Key Project of Anhui Province (Li Jin, Grant no.2024jyxm0215), the Quality Engineering Project of Anhui Province (Li Jin, Grant no.2023sx044) and the Teaching Research Key Project of Anhui University of Chinese Medicine (Li Jin, Grant no.2023xjjy_zd028). This work was supported by the High-level talent introduction project of Anhui University of Chinese Medicine (Liping Sun, Grant no. DT2400000498) The work was supported by the Natural Science Key Project of Anhui Provincial Department of Education (Liping Sun, Grant no. 2024AH051025).The high-level talent introduction project of Anhui University of Chinese Medicine(Jingwen Pan, Grant no. 2024rcyb025).”

Please state what role the funders took in the study.  If the funders had no role, please state: "The funders had no role in study design, data collection and analysis, decision to publish, or preparation of the manuscript."

If this statement is not correct you must amend it as needed.

Please include this amended Role of Funder statement in your cover letter; we will change the online submission form on your behalf.

4. Thank you for stating the following in the Acknowledgments Section of your manuscript:

“The research is supported by the Provincial Teaching Research Key Project of Anhui Province (Li Jin, Grant no.2024jyxm0215), the Quality Engineering Project of Anhui Province (Li Jin, Grant no.2023sx044) and the Teaching Research Key Project of Anhui University of Chinese Medicine (Li Jin, Grant no.2023xjjy_zd028). This work was supported by the High-level talent introduction project of Anhui University of Chinese Medicine (Liping Sun, Grant no. DT2400000498) The work was supported by the Natural Science Key Project of Anhui Provincial Department of Education (Liping Sun, Grant no. 2024AH051025).The high-level talent introduction project of Anhui University of Chinese Medicine(Jingwen Pan, Grant no. 2024rcyb025).”

We note that you have provided funding information that is currently declared in your Funding Statement. However, funding information should not appear in the Acknowledgments section or other areas of your manuscript. We will only publish funding information present in the Funding Statement section of the online submission form.

Please remove any funding-related text from the manuscript and let us know how you would like to update your Funding Statement. Currently, your Funding Statement reads as follows:

“The research is supported by the Provincial Teaching Research Key Project of Anhui Province (Li Jin, Grant no.2024jyxm0215), the Quality Engineering Project of Anhui Province (Li Jin, Grant no.2023sx044) and the Teaching Research Key Project of Anhui University of Chinese Medicine (Li Jin, Grant no.2023xjjy_zd028). This work was supported by the High-level talent introduction project of Anhui University of Chinese Medicine (Liping Sun, Grant no. DT2400000498) The work was supported by the Natural Science Key Project of Anhui Provincial Department of Education (Liping Sun, Grant no. 2024AH051025).The high-level talent introduction project of Anhui University of Chinese Medicine(Jingwen Pan, Grant no. 2024rcyb025).”

Please include your amended statements within your cover letter; we will change the online submission form on your behalf.

5. Your ethics statement should only appear in the Methods section of your manuscript. If your ethics statement is written in any section besides the Methods, please move it to the Methods section and delete it from any other section. Please ensure that your ethics statement is included in your manuscript, as the ethics statement entered into the online submission form will not be published alongside your manuscript.

If the reviewer comments include a recommendation to cite specific previously published works, please review and evaluate these publications to determine whether they are relevant and should be cited. There is no requirement to cite these works unless the editor has indicated otherwise.

[Note: HTML markup is below. Please do not edit.]

Reviewers' comments:

Reviewer's Responses to Questions

**Comments to the Author**

1. Is the manuscript technically sound, and do the data support the conclusions?

Reviewer #1: Partly

Reviewer #2: Yes

**********

2. Has the statistical analysis been performed appropriately and rigorously?

Reviewer #1: No

Reviewer #2: N/A

**********

3. Have the authors made all data underlying the findings in their manuscript fully available?

The PLOS Data policy

Reviewer #1: Yes

Reviewer #2: Yes

**********

4. Is the manuscript presented in an intelligible fashion and written in standard English?

Reviewer #1: No

Reviewer #2: Yes

**********

Reviewer #1: The manuscript presents MFMNet, a novel Tri-Layer Graph Pyramid model equipped with a Cross-Scale Attention Router designed for synergistic drug prediction. The computational architecture is technically sound, and the integration of multiple data modalities is a strong methodological point. Furthermore, the authors are to be commended for their transparent data practices; providing full access to their underlying datasets and model code on GitHub perfectly aligns with open science and data availability standards.

However, to fully satisfy rigorous scientific publishing standards regarding statistical validation and manuscript clarity, several revisions are necessary.

Major Revisions- Statistical Rigor and Methodology

1. Statistical Significance Testing- While Table 2 provides comprehensive performance metrics (AUC, PR_AUC, ACC, etc.) alongside standard deviations, the statistical analysis remains incomplete. The manuscript currently lacks formal statistical significance testing. The authors must include appropriate tests (e.g., paired t-tests or Wilcoxon signed-rank tests) and provide p-values to definitively verify that MFMNet's performance gains over baseline models like DeepDDS and XGBoost are statistically significant rather than due to variance.

2. Model Complexity vs. Sample Size: The authors accurately note that the validation is limited by the size of the dataset, which relies on 668 drug pair evaluations across 8 cell lines. Given the high parameter complexity of the MFMNet architecture—which utilizes a six-layer Transformer and a tri-layer GNN—further discussion is required to explain how the model avoids overfitting this small sample size. Expanding on mitigation strategies beyond early stopping will strengthen the technical credibility of the model.

3. Biological Plausibility of Novel Findings- In Table 1, the top 10 predicted synergistic pairs include two novel combinations (TOPOTECAN & BEZ-235; L778123 & ERLOTINIB) lacking previous literature validation (NA). To better support the conclusion that this model can guide future drug development, the authors should briefly discuss the biological plausibility or potential mechanistic interactions of these specific novel predictions.

Minor Revisions- Manuscript Clarity and Language

The manuscript requires a thorough copyedit to ensure it is written in clear, unambiguous, and standard scientific English. Please address the following typographical, grammatical, and stylistic issues:

1. Typographical & Translation Errors- In the Conclusion section, the architecture is mistakenly referred to as an "adaptive Fri-layer graph pyramid" instead of "Tri-layer". Additionally, the Cross-Scale Attention Router (CSAR) is erroneously referred to as "Caesar's dynamic weight assignment," which appears to be a translation or autocorrect error. In the Ethical standards section, there is a prominent spacing error: "ethi cal".

2. Informal Tone- Portions of the Introduction and Methods sections contain overly casual phrasing and contractions that are not suitable for a formal academic manuscript. Examples include: "They don't block," "And they've also cut," and "What's worse". These should be revised to maintain a professional academic tone.

3. Syntactical Clarity- Certain sentences are awkward and should be rewritten for precision. For example, the sentence "In particular, layers from the 1 to the 1+2 layer add some features..." is highly ambiguous and requires clarification.

Reviewer #2: Comment 1: The novelty of MFMNet should be more clearly distinguished from existing graph neural network-based drug synergy prediction models, particularly DeepDDS and other recent graph-attention architectures.

Comment 2: The manuscript lacks external validation using independent datasets beyond the reported AstraZeneca test set. Validation on additional publicly available drug synergy datasets would strengthen the robustness of the proposed model.

Comment 3: The description of the Tri-Layer Graph Pyramid and Cross-Scale Attention Router is insufficiently detailed. The architectural design and information flow should be explained more clearly to improve reproducibility.

Comment 4: The biological interpretation of the predicted synergistic drug pairs is limited. Additional mechanistic analysis and pathway-level validation would enhance the biological significance of the findings.

Comment 5: The study reports excellent predictive performance; however, statistical significance testing and confidence intervals for comparisons with competing methods should be provided to support the reported improvements.

Comment 6: The model is evaluated mainly through computational experiments. Experimental validation of the newly predicted drug combinations is necessary to demonstrate their translational potential.

Comment 7: The contribution of individual modules, including the Tri-Layer Graph Pyramid, Cross-Scale Attention Router, and feature fusion strategy, should be quantified through more comprehensive ablation studies.

Comment 8: The computational complexity, training time, memory consumption, and scalability of MFMNet are not reported. These aspects should be discussed, particularly for large-scale drug screening applications.

Comment 9: The manuscript should provide greater detail regarding data preprocessing, class imbalance handling, negative sample generation, and hyperparameter optimization to improve reproducibility.

Comment 10: Several figures appear highly stylized and schematic. The figure quality should be improved, and if AI-assisted tools were used to generate the illustrations, this should be disclosed according to the journal's policy.

Comment 11: The Discussion section should include a more critical comparison with recently published state-of-the-art deep learning methods for drug synergy prediction and discuss the limitations of the proposed framework.

Comment 12: The manuscript would benefit from a more detailed discussion of current limitations, including the relatively small dataset, limited biological validation, and potential challenges in generalizing the model to unseen drugs and cancer cell lines.

**********

what does this mean?). If published, this will include your full peer review and any attached files.

If you choose “no”, your identity will remain anonymous but your review may still be made public.

**Do you want your identity to be public for this peer review?**  For information about this choice, including consent withdrawal, please see our Privacy Policy

Reviewer #1: No

Reviewer #2: **Yes:**  NAGMI BANO

**********

[NOTE: If reviewer comments were submitted as an attachment file, they will be attached to this email and accessible via the submission site. Please log into your account, locate the manuscript record, and check for the action link "View Attachments". If this link does not appear, there are no attachment files.]

To ensure your figures meet our technical requirements, please review our figure guidelines: https://journals.plos.org/plosone/s/figures

You may also use PLOS’s free figure tool, NAAS, to help you prepare publication quality figures: https://journals.plos.org/plosone/s/figures#loc-tools-for-figure-preparation.

NAAS will assess whether your figures meet our technical requirements by comparing each figure against our figure specifications.

# Author comment: Author response to Decision Letter 1

**DOI:** 10.1371/journal.pone.0362586.r002

28 Aug 2026

Journal Requirements

1. PLOS ONE style and file-naming requirements

Comment: Please ensure that your manuscript meets PLOS ONE's style requirements, including those for file naming.

Author response: Thank you for this guidance. We revised the title to sentence case, added a short title, removed the postal code from the affiliation, standardized the corresponding-author information, and revised the section headings, figure citations, figure captions, and manuscript formatting to follow the PLOS ONE style requirements. We also removed the standalone Competing interests section from the manuscript because this information is provided through the PLOS submission system; the authors’ declaration that no competing interests exist is retained in the Cover Letter. We will also ensure that the final figure files are named and uploaded in accordance with the journal’s file-naming requirements.

Location in the revised manuscript: Title page, lines 1–10; figure legends, lines 155–157, 433–434, 458–462, 477–479, and 525–529; References, lines 764–938.

2. Code-sharing requirement

Comment: Author-generated code underpinning the findings should be made available without restrictions and in a form that facilitates reproducibility and reuse.

Author response: Thank you for highlighting the code-sharing requirement. The MFMNet code is publicly available in the project repository. For this revision, the repository has been updated to include the final scripts used for the analyses reported in the revised manuscript, including the fold-matched statistical comparisons, ablation experiments, and computational-resource measurements, together with the corresponding outputs and information on the software environment and execution procedures. These materials are provided to facilitate reproducibility and reuse of the reported analyses.

Location in the revised manuscript: Data availability, lines 758–763.

3. Funding disclosure and Role of the Funders

Comment: Please state what role the funders took in the study. If the funders had no role, include the journal-provided statement in the cover letter.

Author response: Thank you for this instruction. We have revised the Funding Statement to reflect the current funding information. The revised statement includes the Provincial Teaching Research Key Project of Anhui Province (Li Jin, Grant No. 2024jyxm0215), the Youth Project of Anhui Provincial Department of Education (Jingwen Pan, Grant No. 2025AHGXZK40314), and the Teaching Research Key Project of Anhui Provincial Department of Education (Liping Sun, Grant No. 2025jyxm0137). The previously listed funding sources that are no longer applicable have been removed. We have also clarified the role of the funders in the revised Cover Letter. The funders provided financial support for the research activities and publication-related expenses but had no role in study design, data collection and analysis, decision to publish, or preparation of the manuscript.

Location in the revised submission: Cover Letter, Funding Statement and Role of the Funders paragraphs on page 1.

4. Remove funding information from Acknowledgments

Comment: Funding information should not appear in the Acknowledgments section or other areas of the manuscript; please remove it and provide the amended statement in the cover letter.

Author response: Thank you for this instruction. We have removed all funding-related information from the Acknowledgments section of the revised manuscript. Funding information is therefore no longer duplicated in the manuscript. The amended Funding Statement and the Role of the Funders statement are provided in the revised Cover Letter for updating the corresponding information in the submission system.

Location in the revised submission: Funding information is provided only in the Cover Letter, page 1; no funding information or Acknowledgments section containing funding information remains in the revised manuscript.

5. Ethics statement location

Comment: The ethics statement should appear only in the Methods section and should be removed from any other section.

Author response: Thank you for this guidance. The revised manuscript now contains the ethics statement only in the Materials and methods section. The separate “Ethical standards” section that appeared outside the Methods section has been removed to avoid duplication and to comply with the journal’s requirement.

Location in the revised manuscript: Materials and methods, “Ethics statement,” lines 184–191.

Reviewer #1

Major Comment 1 — Statistical significance testing

Comment: While Table 2 reports performance metrics with standard deviations, formal statistical significance testing is missing. Appropriate paired tests and p-values should be provided for comparisons with baseline models such as DeepDDS and XGBoost.

Author response: We agree that formal statistical testing is necessary. In the revised manuscript, we performed fold-matched comparisons between MFMNet and DeepDDS-GCN, DeepDDS-GAT, and XGBoost using identical outer-test fold assignments. Two-sided paired t-tests were applied to AUROC and PR-AUC values across matched folds, and Holm correction was performed separately within the AUROC and PR-AUC comparison families. Table 3 reports the mean paired differences, 95% confidence intervals, raw paired p-values, and Holm-adjusted p-values. All six fold-matched comparisons remained statistically significant after Holm correction (adjusted p < 0.05). We also clarified that Table 2 summarizes the initial five-fold cross-validation analysis, whereas Table 3 provides the formal inference under the stricter fold-matched evaluation protocol.

Location in the revised manuscript: “Statistical analysis,” lines 357–379; fold-matched statistical-comparison results, lines 492–504; Table 3 and its explanatory text, lines 505–510.

Major Comment 2 — Model complexity versus sample size

Comment: The reviewer requests a stronger explanation of how overfitting is controlled given the sample size and the complexity of the architecture, beyond early stopping.

Author response: Thank you for raising this important point. We revised both the Materials and methods and Discussion sections to clarify the architecture actually used in the reported experiments and the measures used to reduce the risk of overfitting. For each outer fold, an inner validation subset was used exclusively for model selection and early stopping, while the corresponding outer-test fold remained isolated until the best checkpoint had been selected. In addition to early stopping, the training procedure incorporated dropout, L2 regularization, learning-rate scheduling, and a fixed hyperparameter configuration across all outer folds. We also clarified that these measures reduce, but do not eliminate, the risk of overfitting, particularly because the current dataset contains a limited number of unique drugs and cell lines. Accordingly, the Discussion now distinguishes stability across the evaluated record-level folds from generalization to entirely unseen drugs or cell lines. To make model capacity and computational cost transparent, we additionally report the number of trainable parameters, training time, inference latency and throughput, GPU memory consumption, and scalability across inference batch sizes.

Location in the revised manuscript: “Hyperparameter settings,” lines 211–234; “Computational efficiency and scalability,” lines 568–582; Discussion, lines 604–626 and 693–718.

Major Comment 3 — Biological plausibility of TOPOTECAN + BEZ-235 and L778123 + ERLOTINIB

Comment: The reviewer asks for a discussion of biological plausibility or potential mechanisms for the two novel combinations that lack direct literature validation.

Author response: We appreciate this important suggestion. During revision, we re-evaluated the literature support for all ten top-ranked drug combinations in Table 1. This re-evaluation identified four combinations, rather than two, for which no direct published evidence of the exact drug pair was found: VINBLASTINE–L778123, MITOMYCINE–DASATINIB, TOPOTECAN–BEZ-235, and L778123–ERLOTINIB. We have therefore corrected Table 1 accordingly and added a focused discussion of the biological plausibility of all four combinations, with particular attention to the two combinations highlighted by the reviewer. The discussion is based on the known pharmacological actions and relevant pathway-level evidence for the individual agents and related therapeutic strategies. For TOPOTECAN–BEZ-235, we discuss the potential complementarity between topotecan-associated DNA damage and PI3K–Akt signaling suppression and the inhibition of PI3K/mTOR and DNA-damage repair by BEZ235. For L778123–ERLOTINIB, we discuss the potential complementary effects of prenyltransferase inhibition and EGFR pathway inhibition, together with indirect class-level evidence from related therapeutic strategies. We explicitly distinguish these mechanistic considerations from direct experimental validation and clarify that dedicated in vitro studies are required to determine whether these predicted combinations are genuinely synergistic.

Location in the revised manuscript: Results, lines 406–418 and Table 1 at line 420; Discussion, lines 656–692.

Minor Comment 1 — Typographical and translation errors

Comment: The reviewer identifies “adaptive Fri-layer graph pyramid,” “Caesar's dynamic weight assignment,” and “ethi cal” as errors.

Author response: Thank you for identifying these errors. We have corrected “adaptive Fri-layer graph pyramid” to “adaptive Tri-Layer Graph Pyramid,” replaced the erroneous “Caesar's” wording with the correct Cross-Scale Attention Router (CSAR) terminology, and removed the former standalone “Ethical standards” section outside the Methods section. The ethics statement is now presented only in the Materials and methods section, in accordance with the journal’s requirement. We also reviewed the manuscript for related typographical and terminology inconsistencies and standardized the terminology for the Tri-Layer Graph Pyramid (TGP) and Cross-Scale Attention Router (CSAR) throughout the revised manuscript.

Location in the revised manuscript: Introduction, lines 137–153; “Ethics statement,” lines 184–191; TGP/GCN/GIN/GAT/CSAR methodological descriptions, lines 236–343; Conclusion, lines 726–752.

Minor Comment 2 — Informal tone

Comment: The reviewer notes informal phrases such as “They don't block,” “And they've also cut,” and “What's worse.”

Author response: Thank you for this comment. We have revised the informal and conversational expressions throughout the manuscript to use more precise scientific language. In the Introduction, “They don't block...” was replaced with a statement explaining that single-agent therapies may fail to suppress redundant signaling pathways; “And they've also cut side effects” was revised to describe, more cautiously, the circumstances under which combination strategies may reduce treatment-related adverse effects; and “What's worse” was replaced with “Moreover.” We also revised other conversational expressions in the Results and Discussion sections to maintain a consistent formal academic style throughout the manuscript. In addition, statements regarding model superiority, biological interpretation, and translational relevance were revised where necessary to avoid overly strong or unsupported wording.

Location in the revised manuscript: Introduction, lines 40–74; Results, lines 406–418 and 492–504; Discussion, lines 603–725.

Minor Comment 3 — Syntactical clarity

Comment: The reviewer highlights the sentence “In particular, layers from the l to the l+2 layer add some features...” as ambiguous.

Author response: Thank you for pointing this out. We agree that the sentence was ambiguous. The sentence has been removed from the revised manuscript and replaced by a detailed description of the actual implemented architecture and information flow. The revised Materials and methods section now describes the GCN, GIN, and graph-attention branches separately, including their layer dimensions and processing sequence. The graph-attention branch is explicitly described as being implemented using the GATv2Conv operator. We also describe how the three 128-dimensional branch outputs are concatenated into a 384-dimensional node representation, adaptively reweighted by the Cross-Scale Attention Router, projected to 128 dimensions, and subsequently subjected to masked mean pooling over valid atomic positions before multimodal classification. These revisions ensure that the methodological description corresponds to the implemented model rather than relying on the previous ambiguous layer-to-layer wording.

Location in the revised manuscript: “GCN,” “GIN,” and “GAT,” lines 236–294; “Adaptive feature fusion module,” lines 318–343; “Classification module,” lines 344–355.

Reviewer #2

Comment 1 — Novelty relative to DeepDDS and recent graph-attention models

Comment: The novelty of MFMNet should be more clearly distinguished from existing GNN-based drug-synergy models, particularly DeepDDS and recent graph-attention architectures.

Author response: Thank you for this important suggestion. We have revised the Introduction to more clearly position MFMNet relative to DeepDDS and more recent graph- and attention-based drug-synergy prediction models. Specifically, we added a focused comparison with DeepDDS and representative recent approaches using graph attention, heterogeneous graph attention, drug-pair attention, and cross-modal attention. The revised discussion clarifies that the contribution of MFMNet does not lie simply in introducing an attention mechanism. Instead, MFMNet combines complementary molecular representations generated by graph convolutional, graph isomorphism, and graph-attention operations within the Tri-Layer Graph Pyramid (TGP), and uses the Cross-Scale Attention Router (CSAR) to coordinate information across these representations before integration with cell-line features. We have therefore clarified that the principal architectural distinction of MFMNet is its coordinated multi-scale graph representation and cross-scale routing strategy.

Location in the revised manuscript: Introduction, lines 118–153.

Comment 2 — Additional independent external validation

Comment: Validation on additional publicly available drug-synergy datasets beyond the reported AstraZeneca test set is recommended.

Author response: We thank the reviewer for this important suggestion. We added cross-dataset evaluations using two publicly available drug-combination datasets beyond the model-development data. To avoid selecting an external dataset on the basis of model performance, candidate DrugComb studies were screened before inference according to predefined criteria, including explicit study provenance, compatibility with the available molecular-graph and cell-line input features, removal of exact O’Neil drug-pair–cell-line overlaps, and availability of both outcome classes under the fixed Loewe-based classification rule. The source-resolved FRIEDMAN study satisfied these criteria. After removal of exact O’Neil overlaps and exclusion of records with Loewe scores between 0 and 10, 39 external records remained, comprising 9 synergistic and 30 non-synergistic combinations across 10 drugs and 5 cell lines.

During preparation of the external evaluation, we found that the supplied 768-dimensional auxiliary drug embeddings were record-dependent and could not be reconstructed consistently for independent drug-pair–cell-line records. Arbitrary external imputation of these features was therefore avoided. The additional evaluations used the pre-defined embedding-independent MFMNet configuration, which retained the GCN, GIN, and GATv2 molecular-graph branches, the Cross-Scale Attention Router, the cell-line encoder, and the downstream classifier. Five checkpoints selected exclusively using the O’Neil training and validation data were frozen before external inference and combined using fixed equal weights. No external labels were used for checkpoint selection, threshold tuning, feature construction, or ensemble weighting.

On the FRIEDMAN external study, this configuration achieved an AUROC of 0.7704 and a PR-AUC of 0.6602, with bootstrap 95% confidence intervals of 0.5031–0.9500 and 0.2544–0.9028, respectively. Because only 39 classifiable external records were available, we interpret this result cautiously as supportive evidence of cross-study transferability rather than definitive evidence of broad generalization.

We additionally evaluated the same frozen configuration on 864 NCI-ALMANAC records after removal of exact O’Neil drug-pair–cell-line overlaps. The resulting AUROC was 0.5407 and the PR-AUC was 0.2455, with 95% confidence intervals of 0.4817–0.5975 and 0.1928–0.3053, respectively. We retained this less favorable external result in the revised analysis because it provides important information regarding cross-dataset distribution and endpoint shift. In particular, NCI-ALMANAC ComboScore and the O’Neil Loewe score are not identical response measures. The revised manuscript now reports both the supportive FRIEDMAN result and the more challenging NCI-ALMANAC result and explicitly limits the corresponding generalization claims.

Location in the revised manuscript: “Additional external validation,” lines 380–401; “Additional cross-dataset external evaluation,” lines 591–602; Discussion, lines 643–655 and 693–718.

Comment 3 — More detailed TGP and CSAR architecture/information flow

Comment: The architectural design and information flow of the Tri-Layer Graph Pyramid and Cross-Scale Attention Router should be explained more clearly for reproducibility.

Author response: Thank you for this comment. We substantially expanded the Materials and methods section to provide a stepwise description of the implemented TGP and CSAR architecture and information flow. The revised manuscript separately describes the GCN, GIN, and graph-attention branches, including their layer dimensions and processing sequences. The graph-attention branch is explicitly described as being implemented using the GATv2Conv operator. The 128-dimensional node representations produced by the three graph branches are concatenated into a 384-dimensional representation, which is adaptively reweighted by the CSAR, projected to 128 dimensions, and subjected to masked mean pooling over valid atomic positions. The resulting graph representations are then integrated with the two drug embeddings and the cell-line transcriptomic representation to form the input to the final classifier. These revisions align the methodological description with the implemented architecture and provide a clearer account of the complete information flow for reproducibility.

Location in the revised manuscript: “GCN,” “GIN,” and “GAT,” lines 236–294; “Adaptive feature fusion module,” lines 318–343; “Classification module,” lines 344–355.

Comment 4 — Biological interpretation and pathway-level validation

Comment: Additional mechanistic analysis and pathway-level validation would strengthen the biological significance of the predicted synergistic drug pairs.

Author response: We agree that stronger biological interpretation improves the biological significance of the predicted combinations. Because no direct pathway-level experiments were performed in the present study, we did not present the proposed mechanisms as experimentally validated pathways. Instead, we added a literature-supported mechanistic discussion of the top-ranked combinations for which no direct evidence of the exact drug pair was identified. In particular, the revised Discussion examines plausible biological interactions for VINBLASTINE–L778123, MITOMYCINE–DASATINIB, TOPOTECAN–BEZ-235, and L778123–ERLOTINIB on the basis of the established pharmacological actions of the individual agents and relevant pathway-level evidence. For TOPOTECAN–BEZ-235, we discuss the potential complementarity between topotecan-associated DNA damage and PI3K–Akt signaling suppression and the PI3K/mTOR and DNA-repair inhibitory effects of BEZ235. For L778123–ERLOTINIB, we discuss the potential complementarity between prenyltransferase inhibition and EGFR pathway inhibition. We explicitly characterize these explanations as testable mechanistic hypotheses rather than direct pathway-level validation and state that dedicated biological experiments will be required for confirmation.

Location in the revised manuscript: Results, lines 406–418 and Table 1 at line 420; Discussion, lines 656–692.

Comment 5 — Statistical significance and confidence intervals

Comment: The reviewer requests statistical significance testing and confidence intervals for comparisons with competing methods.

Author response: Thank you for this important point. We added a dedicated statistical-analysis subsection and performed fold-matched comparisons using identical outer-test fold assignments. MFMNet was compared with DeepDDS-GCN, DeepDDS-GAT, and XGBoost for AUROC and PR-AUC using two-sided paired t-tests. The revised manuscript reports mean paired differences, 95% confidence intervals, raw paired p-values, and Holm-adjusted p-values in Table 3, with Holm correction applied separately to the AUROC and PR-AUC comparison families. All six fold-matched comparisons remained statistically significant after correction (adjusted p < 0.05). We also distinguish the descriptive initial five-fold cross-validation results in Table 2 from the stricter fold-matched statistical comparison in Table 3.

Location in the revised manuscript: “Statistical analysis,” lines 357–379; fold-matched statistical-comparison results, lines 492–504; Table 3 and its explanatory text, lines 505–510.

Comment 6 — Experimental validation of newly predicted combinations

Comment: The reviewer states that experimental validation of newly predicted drug combinations is necessary to demonstrate translational potential.

Author response: We appreciate the reviewer's emphasis on experimental validation and agree that wet-lab validation is important for establishing the translational relevance of newly predicted drug combinations. The present study is computational in scope, and no new in vitro experiments were performed in this revision. We have therefore revised the manuscript to avoid presenting the newly predicted combinations as experimentally validated synergies. The Results and Discussion now distinguish literature-supported mechanistic hypotheses from direct biological validation, and the manuscript explicitly states that dedicated in vitro studies will be required to determine whether these predicted interactions are genuinely synergistic. We also moderated statements concerning clinical and translational potential in the Abstract, Discussion, and Conclusion and explicitly identified the absence of wet-lab validation as a limitation of the current study. Accordingly, the predicted combinations are presented as candidates for subsequent experimental investigation rather than as experimentally confirmed therapeutic combinations.

Location in the revised manuscript: Abstract, lines 12–36; Results, lines 406–418 and Table 1 at line 420; Discussion, lines 656–718; Conclusion, lines 726–752.

Comment 7 — Comprehensive ablation studies

Comment: The reviewer requests quantitative evaluation of the contributions of the Tri-Layer Graph Pyramid, Cross-Scale Attention Router, and feature-fusion strategy.

Author response: Thank you for this suggestion. We have added a controlled ablation analysis to quantitatively evaluate the contributions of the Tri-Layer Graph Pyramid (TGP), Cross-Scale Attention Router (CSAR), and graph-feature fusion strategy. All variants were evaluated using the same five outer-test folds, inner-validation procedure, optimization budget, stopping rule, and controlled fold-specific initialization.

Specifically, we compared the full MFMNet with variants removing the GCN, GIN, or GAT branch, as well as variants replacing CSAR with unweighted mean fusion or simple concatenation followed by linear projection. The full model achieved an AUROC of 0.9227 ± 0.0055 and a PR-AUC of 0.9127 ± 0.0084. Removing GCN and GIN resulted in mean AUROCs of 0.9213 ± 0.0039 and 0.9206 ± 0.0024, respectively, whereas removing GAT yielded a similar AUROC of 0.9229 ± 0.0032. Replacing CSAR with mean fusion reduced the mean AUROC to 0.9192 ± 0.0041, while simple concatenation yielded 0.9182 ± 0.0010. The corresponding mean AUROC differences in favor of the full model were 0.0035 and 0.0045 for mean fusion and simple concatenation, respectively. Paired comparisons were performed across identical folds with Holm correction; none of the primary ablation comparisons reached statistical significance after multiple-comparison correction.

We further added branch-level diagnostic analyses to examine complementarity among the GCN, GIN, and GAT branches. Pairwise prediction disagreement rates were approximately 8.7%–9.1%, each branch uniquely classified a subset of samples correctly, and pairwise representation similarities were low overall. These findings indicate that the three graph encoders capture substantially non-redundant and partially complementary molecular information, although their marginal contributions to aggregate predictive performance are heterogeneous. We have revised the manuscript accordingly and have avoided interpreting the non-significant ablation differences as statistically significant improvements.

Location in the revised manuscript: “Statistical analysis,” lines 366–379; “Controlled ablation analysis of TGP and CSAR,” lines 530–567; Discussion, lines 719–725.

Comment 8 — Computational complexity, time, memory, and scalability

Comment: The reviewer requests computational complexity, training time, memory consumption, and scalability information.

Author response: Thank you for this comment. We added a dedicated computational-efficiency and scalability analysis using the revised model implementation. The full MFMNet backbone contained approximately 2.65 million trainable parameters. Across the five outer folds, the mean active training time was approximately 27.6 min per fold and the mean peak accelerator memory usage during training was approximately 565.3 MB. At an inference batch size of 64, the mean inference time was approximately 1.00 ms per sample, corresponding to a throughput of approximately 1,003 samples/s. When the inference batch size was increased from 32 to 512, throughput increased from approximately 804 to 1,256 samples/s, while peak accelerator memory usage increased from approximately 123 MB to 1.05 GB. These results were added to make the computational requirements and scalability of MFMNet more transparent.

Location in the revised manuscript: “Computational efficiency and scalability,” lines 568–582.

Comment 9 — Reproducibility details

Comment: More detail is requested for data preprocessing, class-imbalance handling, negative-sample generation, and hyperparameter optimization.

Author response: We agree and have expanded the Materials and methods section to provide a more explicit account of the data-processing and training procedures. The revised manuscript now reports the dataset size, numbers of unique drugs and cell lines, synergy-score criteria used for binary-label construction, class distribution, and molecular-graph preprocessing procedure. We clarify that no additional negative samples were synthetically generated and that the original class distribution was retained without oversampling, undersampling, or class-weighted loss. We also describe the five-fold outer-test partitioning, inner validation subsets used for model selection, fixed random-seed strategy, optimization settings, learning-rate scheduling, regularization, and early-stopping procedure. The same hyperparameter configuration was maintained across all outer folds, and no fold-specific hyperparameter tuning was performed using the corresponding outer-test data. The accompanying public repository also provides the software and execution information used for the revised experiments to facilitate reproducibility.

Location in the revised manuscript: “Data source and label definition,” lines 159–182; “Data preprocessing,” lines 193–209; “Hyperparameter settings,” lines 211–234.

Comment 10 — Figure quality and AI-assisted figure generation

Comment: Several figures appear highly stylized and schematic; figure quality should be improved, and AI-assisted illustration generation should be disclosed if applicable.

Author response: Thank you for this comment. We revised the figures to improve scientific presentation and readability by simplifying schematic elements, standardizing fonts, labels, line styles, and panel formatting, and regenerating quantitative plots from the verified experimental results. Figure 1 was reconstructed as an editable schematic so that the TGP, CSAR, masked pooling, multimodal integration, and classifier information flow correspond directly to the implemented architecture. The quantitative performance figures were also updated to match the final reported results. AI-assisted tools were used during preliminary figure drafting, and Grammarly was used solely for language proofreading. The final figures were reconstructed and verified by the authors against the implemented model and experimental results.

Location in the revised manuscript: Fig 1 legend, lines 155–157; Fig 2 legend, lines 433–434; Fig 3 legend, lines 458–462; Fig 4 legend, lines 477–479; Fig 5 legend, lines 525–529.

Comment 11 — Critical comparison with recent state-of-the-art methods

Comment: The Discussion should include a more critical comparison with recent state-of-the-art deep-learning methods and discuss the limitations of MFMNet.

Author response: Thank you for this suggestion. We have revised the Discussion to include a concise comparison with recent graph- and attention-based drug-synergy prediction methods, including DeepDDS, AttenSyn, HANSynergy, and SynergyX. The revised text highlights the distinction of MFMNet in jointly integrating heterogeneous GCN, GIN, and GAT representations through TGP and coordinating these representations using CSAR. We also clarify that, although MFMNet showed statistically significant improvements over the fold-matched DeepDDS variants in the present study, direct numerical comparisons with other recent methods should be interpreted cautiously because of differences in datasets and evaluation protocols. The limitations of MFMNet are further discussed separately in the revised Discussion.

Location in the revised manuscript: Discussion, lines 627–642.

Comment 12 — Limitations and generalization to unseen drugs/cell lines

Comment: The reviewer requests a fuller limitations discussion, including the relatively small dataset, limited biological validation, and challenges in generalizing to unseen drugs and cancer cell lines.

Author response: We agree and have substantially expanded the limitations discussion. The revised manuscript now explicitly states that, although the analyzed dataset contains 13,243 labeled drug-combination records, it includes only 38 unique drugs and 31 cancer cell lines, thereby limiting the chemical and cellular diversity represented in the study. We further clarify that the current five-fold record-level evaluation does not specifically assess generalization to entirely unseen drugs or unseen cell lines and that stability across the evaluated outer folds should not be interpreted as evidence of out-of-distribution generalization. The revised Discussion also identifies the absence of direct pathway-level experimental validation and wet-lab validation of the newly predicted combinations as important limitations. In addition, we note that the current model primarily uses molecular graphs, drug embeddings, and cell-line transcriptomic features without broader multi-omics or additional drug-metabolism information. Translational claims have therefore been moderated, and validation on broader datasets and under unseen-drug and unseen-cell-line settings is identified as an important direction for future work.

Location in the revised manuscript: Discussion, lines 643–655 and 693–718.

Attachment Submitted filename: Response to Reviewers.doc

# Peer review: Decision Letter 1

**DOI:** 10.1371/journal.pone.0362586.r003

**Authors:** Junhuang Jiang

20 Oct 2026

Dear Dr. Jin,

Thank you for submitting your manuscript to PLOS One. After careful consideration, we feel that it has merit but does not fully meet PLOS One’s publication criteria as it currently stands. Therefore, we invite you to submit a revised version of the manuscript that addresses the points raised during the review process.

Please submit your revised manuscript by Dec 04 2026 11:59PM. If you will need more time than this to complete your revisions, please reply to this message or contact the journal office at plosone@plos.org. When you're ready to submit your revision, log on to https://www.editorialmanager.com/pone/ and select the 'Submissions Needing Revision' folder to locate your manuscript file.

- A letter that responds to each point raised by the academic editor and reviewer(s). You should upload this letter as a separate file labeled 'Response to Reviewers'.
- A marked-up copy of your manuscript that highlights changes made to the original version. You should upload this as a separate file labeled 'Revised Manuscript with Track Changes'.
- An unmarked version of your revised paper without tracked changes. You should upload this as a separate file labeled 'Manuscript'.

If you would like to make changes to your financial disclosure, please include your updated statement in your cover letter. Guidelines for resubmitting your figure files are available below the reviewer comments at the end of this letter.

If applicable, we recommend that you deposit your laboratory protocols in protocols.io to enhance the reproducibility of your results. Protocols.io assigns your protocol its own identifier (DOI) so that it can be cited independently in the future. For instructions see: https://journals.plos.org/plosone/s/submission-guidelines#loc-laboratory-protocols. Additionally, PLOS One offers an option for publishing peer-reviewed Lab Protocol articles, which describe protocols hosted on protocols.io. Read more information on sharing protocols at https://plos.org/protocols?utm_medium=editorial-email&utm_source=authorletters&utm_campaign=protocols.

As the corresponding author, your ORCID iD is verified in the submission system and will appear in the published article. PLOS supports the use of ORCID, and we encourage all coauthors to register for an ORCID iD and use it as well. Please encourage your coauthors to verify their ORCID iD within the submission system before final acceptance, as unverified ORCID iDs will not appear in the published article. *Only*  the individual author can complete the verification step; PLOS staff *cannot*  verify ORCID iDs on behalf of authors.

We look forward to receiving your revised manuscript.

Kind regards,

Junhuang Jiang

Academic Editor

PLOS One

Journal Requirements:

If the reviewer comments include a recommendation to cite specific previously published works, please review and evaluate these publications to determine whether they are relevant and should be cited. There is no requirement to cite these works unless the editor has indicated otherwise.

Please review your reference list to ensure that it is complete and correct. If you have cited papers that have been retracted, please include the rationale for doing so in the manuscript text, or remove these references and replace them with relevant current references. Any changes to the reference list should be mentioned in the rebuttal letter that accompanies your revised manuscript. If you need to cite a retracted article, indicate the article’s retracted status in the References list and also include a citation and full reference for the retraction notice.

[Note: HTML markup is below. Please do not edit.]

Reviewers' comments:

Reviewer's Responses to Questions

**Comments to the Author**

Reviewer #1: (No Response)

Reviewer #3: All comments have been addressed

**********

2. Is the manuscript technically sound, and do the data support the conclusions?

Reviewer #1: Yes

Reviewer #3: Yes

**********

3. Has the statistical analysis been performed appropriately and rigorously?

Reviewer #1: Yes

Reviewer #3: Yes

**********

4. Have the authors made all data underlying the findings in their manuscript fully available?

The PLOS Data policy

Reviewer #1: Yes

Reviewer #3: Yes

**********

5. Is the manuscript presented in an intelligible fashion and written in standard English?

Reviewer #1: Yes

Reviewer #3: Yes

**********

Reviewer #1: The research is technically sound and addresses a significant bottleneck in oncology drug discovery. However, a few areas regarding statistical clarity and textual presentation require minor revisions before publication.

Major Comments (Methodology & Statistical Rigor)

Cross-Validation and Data Leakage: The authors appropriately utilize 5-fold cross-validation to evaluate model performance, reporting an average AUC of 0.929. To ensure absolute statistical rigor, the authors should explicitly confirm in the Methods section that hyperparameter tuning (mentioned in section 2.3) was conducted strictly within the training folds (e.g., using nested cross-validation) and that the test folds remained entirely unseen to prevent data leakage.

Data Availability Statement: The authors have laudably provided GitHub links for the drug-cell combination dataset, model code, training weights, and experimental scripts under the MIT License. To fully comply with open science best practices, please ensure that the repository contains an explicit README.md with step-by-step instructions on how to replicate the specific figures (e.g., Figure 2 heatmaps and Figure 3 scatter plots) presented in the manuscript.

Performance Metrics: Table 2 provides a comprehensive multi-metric comparison featuring standard deviations (e.g., AUC 0.9301 ± 0.030 for MFMNet). It would strengthen the statistical analysis to briefly state whether the differences between MFMNet and the second-best model (DeepDDS) are statistically significant (e.g., via a paired t-test across the folds).

Minor Comments (Manuscript Clarity & Formatting)

Language and Phrasing: While the manuscript is generally intelligible, the Introduction contains some colloquial or awkward phrasing that should be revised for standard academic tone. For instance, the phrase "makes single-agent treatment an automatic failure by default" is overly absolute and informal. Similarly, "Such is an age-old relationship between synergy and medicine" should be rephrased for scientific objectivity.

Typographical Consistency: In Table 1, the alignment and hyphenation of drug names (e.g., "VINBLASTIN E", "DASATINI B") appear fragmented due to formatting. Please ensure standard nomenclature formatting in the final tables.

Figure Clarity: Figure 4 contains multiple dense histograms and ROC curves. Ensuring high-resolution vector graphics are uploaded during the revision will help readers distinguish the overlapping distributions clearly.

Reviewer #3: Recommendation: Accept with Minor Revisions.

Editorial Justification: The authors have delivered an exemplary revision that successfully addresses all primary concerns raised during the initial review. Key strengths of the revision include:

Implementation of fold-matched statistical testing with Holm correction, confirming genuine baseline superiority ( $p_{adj} < 0.05$ ).

Complete resolution of architectural ambiguities regarding TGP and CSAR dimension transformations.

Transparent reporting of negative and challenging results, including non-significant ablation $p$ -values and performance drops on NCI-ALMANAC due to endpoint shift.

Full compliance with code and data sharing policies via public GitHub repositories under an MIT License.The manuscript is suitable for publication in PLOS ONE following minor textual and repository adjustments.

5.2 Actionable Directive Comments for the Authors

Prior to formal typesetting and publication, the authors must address the following minor action items:

Refine Generalization Language: Review the Abstract and Conclusion to ensure all assertions strictly reflect record-level cross-validation results, explicitly noting that generalization to unseen drugs and cell lines requires further study.

Code Repository Maintenance: Ensure that execution scripts, environment specifications, and frozen checkpoint loaders for the embedding-independent model variant used in the FRIEDMAN and NCI-ALMANAC evaluations remain permanently archived in the primary GitHub repository (https://github.com/wuyababy/MFMNET-2/tree/main).

Figure Text Verification: Verify that all reconstructed figures (Figs 1–5) maintain uniform font sizes, uncompressed high-resolution panel renderings, and clear typography in accordance with PLOS ONE final submission specifications.

**********

what does this mean?). If published, this will include your full peer review and any attached files.

If you choose “no”, your identity will remain anonymous but your review may still be made public.

**Do you want your identity to be public for this peer review?**  For information about this choice, including consent withdrawal, please see our Privacy Policy

Reviewer #1: No

Reviewer #3: **Yes:**  Joshua Banda

**********

[NOTE: If reviewer comments were submitted as an attachment file, they will be attached to this email and accessible via the submission site. Please log into your account, locate the manuscript record, and check for the action link "View Attachments". If this link does not appear, there are no attachment files.]

To ensure your figures meet our technical requirements, please review our figure guidelines: https://journals.plos.org/plosone/s/figures

You may also use PLOS’s free figure tool, NAAS, to help you prepare publication quality figures: https://journals.plos.org/plosone/s/figures#loc-tools-for-figure-preparation.

NAAS will assess whether your figures meet our technical requirements by comparing each figure against our figure specifications.

# Author comment: Author response to Decision Letter 2

**DOI:** 10.1371/journal.pone.0362586.r004

25 Oct 2026

Comments to the reviewers and all changes with the draft are summarized below

Reviewer #1

“The research is technically sound and addresses a significant bottleneck in oncology drug discovery. However, a few areas regarding statistical clarity and textual presentation require minor revisions before publication.”

Major Comment 1 — Cross-validation and data leakage

Comment: The authors appropriately utilize 5-fold cross-validation to evaluate model performance, reporting an average AUC of 0.929. To ensure absolute statistical rigor, the authors should explicitly confirm in the Methods section that hyperparameter tuning (mentioned in section 2.3) was conducted strictly within the training folds (e.g., using nested cross-validation) and that the test folds remained entirely unseen to prevent data leakage.

Author response: We agree, and an explicit statement has been added at the end of the “Hyperparameter settings” section: “Hyperparameter tuning and model selection were therefore performed exclusively on the training and validation data of each outer fold, and no information from the outer-test folds—including records, labels, or summary statistics—was used at any stage of model development; each outer-test fold was evaluated exactly once, after the final checkpoint had been frozen.” The cross-validation architecture—an outer five-fold partition with a stratified inner-validation split, early stopping on validation AUROC only, and post hoc evaluation of the untouched outer-test fold—corresponds exactly to the protocol the reviewer describes, and the new sentence states these guarantees explicitly in the Methods.

Location in the revised manuscript: Materials and methods, “Hyperparameter settings,” lines 240–244.

Major Comment 2 — Data Availability Statement (README for figure replication)

Comment: The authors have laudably provided GitHub links for the drug-cell combination dataset, model code, training weights, and experimental scripts under the MIT License. To fully comply with open science best practices, please ensure that the repository contains an explicit README.md with step-by-step instructions on how to replicate the specific figures (e.g., Figure 2 heatmaps and Figure 3 scatter plots) presented in the manuscript.

Author response: The repository now contains a README.md with (i) environment installation instructions, (ii) download links for the drug–cell-line combination dataset, and (iii) step-by-step commands and scripts for reproducing the manuscript's data-driven figures: Figure 2 (atomic-feature similarity heatmaps) is regenerated directly from the released fold checkpoints; Figures 3–5 are regenerated from the released fold-level metric and per-sample prediction CSVs, with the commands and the file sources of every reported value documented in the README; and Figure 1 is a schematic architecture diagram and is annotated as such. The README and the accompanying scripts, per-fold metrics, per-sample predictions, and frozen checkpoints were added to the repository in this revision: https://github.com/wuyababy/MFMNET-2/tree/main (commit a6441827, https://github.com/wuyababy/MFMNET-2/commit/a644182723cb6423e6c0dc52c40390d880bfd667).

Location in the revised manuscript: Data availability, lines 797–799.

Major Comment 3 — Performance Metrics (significance versus DeepDDS)

Comment: Table 2 provides a comprehensive multi-metric comparison featuring standard deviations (e.g., AUC 0.9301 ± 0.030 for MFMNet). It would strengthen the statistical analysis to briefly state whether the differences between MFMNet and the second-best model (DeepDDS) are statistically significant (e.g., via a paired t-test across the folds).

Author response: We agree that stating the DeepDDS-specific results explicitly strengthens the statistical analysis. The fold-matched paired t-tests with Holm correction are reported in Table 3 (two-sided, computed across the five identical outer-test folds, with correction applied separately within the AUROC and PR-AUC comparison families). To make the key result explicit, the following sentence has been added immediately after the existing significance statement in the Results section: “In particular, MFMNet significantly outperformed both DeepDDS variants on both metrics under Holm-adjusted paired t-tests: p = 0.0099 (AUROC) and 0.0075 (PR-AUC) versus DeepDDS-GCN, and p = 0.0130 (AUROC) and 0.0332 (PR-AUC) versus DeepDDS-GAT.”

Location in the revised manuscript: Results, “Multi-metric comparative experiments using state-of-the-art methods,” lines 529–533.

Minor Comment 1 — Language and phrasing

Comment: While the manuscript is generally intelligible, the Introduction contains some colloquial or awkward phrasing that should be revised for standard academic tone. For instance, the phrase “makes single-agent treatment an automatic failure by default” is overly absolute and informal. Similarly, “Such is an age-old relationship between synergy and medicine” should be rephrased for scientific objectivity.

Author response: We agree that such phrasing is inappropriate for a scientific manuscript. Both sentences were already removed during the first revision and are absent from the clean manuscript; the passages noted by the reviewer correspond to the marked-up revision copy, in which they appear only as tracked deletions. The corresponding passage now reads: “Single-agent therapies may fail to suppress redundant signaling pathways; for example, the effects of EGFR inhibition in colorectal cancer can be attenuated by compensatory activation of MET signaling, contributing to treatment resistance and relapse [2].” We have re-examined the Introduction to ensure a consistent academic tone throughout.

Location in the revised manuscript: Introduction, lines 46–49.

Minor Comment 2 — Typographical consistency in Table 1

Comment: In Table 1, the alignment and hyphenation of drug names (e.g., “VINBLASTIN E”, “DASATINI B”) appear fragmented due to formatting. Please ensure standard nomenclature formatting in the final table

Author response: The drug names stored in the manuscript text are spelled correctly (e.g., VINBLASTINE, DASATINIB); the fragmentation the reviewer observed arose from column-width text wrapping in the rendered PDF, where long names were broken mid-word. In the revised manuscript we have (i) fixed the table layout (fixed-layout table with widened “Drug A” (4.32 cm) and “Drug B” (3.97 cm) columns) so that every drug name is displayed intact on a single line, and (ii) verified all drug names against standard nomenclature. During this verification we identified and corrected one genuine spelling error, “MITOMYCINE” → “MITOMYCINC”, in Table 1 and in the corresponding Results paragraph; the corrected spelling follows the drug's standard name (mitomycin C) as used in the source dataset and in reference [42].

Location in the revised manuscript: Table 1, line 436; Results, lines 690–700.

Minor Comment 3 — Figure clarity (Figure 4)

Comment: Figure 4 contains multiple dense histograms and ROC curves. Ensuring high-resolution vector graphics are uploaded during the revision will help readers distinguish the overlapping distributions clearly.

Author response: Figure 4 has been re-exported as an uncompressed high-resolution (300 dpi) TIFF with flattened RGB output and clear, uniform typography, so that the overlapping distributions can be distinguished clearly. All other figures (Figs 1–5) were re-examined and re-exported at ≥300 dpi with uniform font sizes, consistent with PLOS ONE figure specifications.

Location in the revised manuscript: Fig 4 legend, lines 502–504.

Reviewer #3

“Recommendation: Accept with Minor Revisions.” We thank the reviewer for the positive assessment and the concrete action items, which are addressed below.

Comment 1 — Refine generalization language

Comment: Review the Abstract and Conclusion to ensure all assertions strictly reflect record-level cross-validation results, explicitly noting that generalization to unseen drugs and cell lines requires further study.

Author response: We have revised the Abstract and Conclusion accordingly. Abstract, Conclusions (now reads): “The results support its use for computational prioritization of candidate synergistic drug combinations. All reported performance estimates refer to record-level five-fold cross-validation within the O'Neil dataset; generalization to entirely unseen drugs and cell lines requires further study.” Conclusion (added): “Because all performance estimates were obtained by record-level cross-validation within a single dataset, generalization to entirely unseen drugs and cell lines remains to be established in future work.”

Location in the revised manuscript: Abstract, lines 33–37; Conclusion, lines 783–785.

Comment 2 — Code repository maintenance

Comment: Ensure that execution scripts, environment specifications, and frozen checkpoint loaders for the embedding-independent model variant used in the FRIEDMAN and NCI-ALMANAC evaluations remain permanently archived in the primary GitHub repository (https://github.com/wuyababy/MFMNET-2/tree/main).

Author response: The execution scripts, environment specifications (requirements.txt), and the frozen checkpoints of the embedding-independent model variant (mfmnet_no_drug_embedding_fold1–5.pt) are permanently archived in the primary repository: https://github.com/wuyababy/MFMNET-2/tree/main (commit a6441827).

Location in the revised manuscript: Data availability, lines 797–799.

Comment 3 — Figure text verification

Comment: Verify that all reconstructed figures (Figs 1–5) maintain uniform font sizes, uncompressed high-resolution panel renderings, and clear typography in accordance with PLOS ONE final submission specifications.

Author response: All reconstructed figures (Figs 1–5) were re-exported with uniform font sizes, uncompressed high-resolution rendering, and clear typography in accordance with the PLOS ONE final submission specifications.

Location in the revised manuscript: Fig 1 legend, line 161; Fig 5 legend, lines 556–560; and the corresponding legends of Figs 2–4.

Attachment Submitted filename: Response_to_Reviewers_auresp_2.doc

# Editor report: Decision Letter 2

**DOI:** 10.1371/journal.pone.0362586.r005

**Authors:** Junhuang Jiang

28 Oct 2026

MFMNet: Adaptive tri-layer graph pyramid with cross-scale attention router for synergistic drug prediction

PONE-D-26-19304R2

Dear Dr. Jin,

We’re pleased to inform you that your manuscript has been judged scientifically suitable for publication and will be formally accepted for publication once it meets all outstanding technical requirements.

Within one week, you’ll receive an e-mail detailing the required amendments. When these have been addressed, you’ll receive a formal acceptance letter and your manuscript will be scheduled for publication.

An invoice will be generated when your article is formally accepted. Please note, if your institution has a publishing partnership with PLOS and your article meets the relevant criteria, all or part of your publication costs will be covered. Please make sure your user information is up-to-date by logging into Editorial Manager at Editorial Manager® and clicking the ‘Update My Information' link at the top of the page. For questions related to billing, please contact billing support.

If your institution or institutions have a press office, please notify them about your upcoming paper to help maximize its impact. If they’ll be preparing press materials, please inform our press team as soon as possible -- no later than 48 hours after receiving the formal acceptance. Your manuscript will remain under strict press embargo until 2 pm Eastern Time on the date of publication. For more information, please contact onepress@plos.org.

Kind regards,

Junhuang Jiang

Academic Editor

PLOS One

Additional Editor Comments (optional):

Reviewers' comments:

# Editor report: Acceptance letter

**DOI:** 10.1371/journal.pone.0362586.r006

**Authors:** Junhuang Jiang

PONE-D-26-19304R2

PLOS One

Dear Dr. Jin,

I'm pleased to inform you that your manuscript has been deemed suitable for publication in PLOS One. Congratulations! Your manuscript is now being handed over to our production team.

At this stage, our production department will prepare your paper for publication. This includes ensuring the following:

* All references, tables, and figures are properly cited

* All relevant supporting information is included in the manuscript submission,

* There are no issues that prevent the paper from being properly typeset

You will receive further instructions from the production team, including instructions on how to review your proof when it is ready. Please keep in mind that we are working through a large volume of accepted articles, so please give us a few days to review your paper and let you know the next and final steps.

Lastly, if your institution or institutions have a press office, please let them know about your upcoming paper now to help maximize its impact. If they'll be preparing press materials, please inform our press team within the next 48 hours. Your manuscript will remain under strict press embargo until 2 pm Eastern Time on the date of publication. For more information, please contact onepress@plos.org.

You will receive an invoice from PLOS for your publication fee after your manuscript has reached the completed accept phase. If you receive an email requesting payment before acceptance or for any other service, this may be a phishing scheme. Learn how to identify phishing emails and protect your accounts at https://explore.plos.org/phishing.

If we can help with anything else, please email us at customercare@plos.org.

Thank you for submitting your work to PLOS One and supporting open access.

Kind regards,

PLOS One Editorial Office Staff

on behalf of

Dr. Junhuang Jiang

Academic Editor

PLOS One
